# Reducing GBA2 Activity Ameliorates Neuropathology in Niemann-Pick Type C Mice

**DOI:** 10.1371/journal.pone.0135889

**Published:** 2015-08-14

**Authors:** André R. A. Marques, Jan Aten, Roelof Ottenhoff, Cindy P. A. A. van Roomen, Daniela Herrera Moro, Nike Claessen, María Fernanda Vinueza Veloz, Kuikui Zhou, Zhanmin Lin, Mina Mirzaian, Rolf G. Boot, Chris I. De Zeeuw, Herman S. Overkleeft, Yildiz Yildiz, Johannes M. F. G. Aerts

**Affiliations:** 1 Department of Medical Biochemistry, Academic Medical Center, 1105 AZ, Amsterdam, The Netherlands; 2 Department of Pathology, Academic Medical Center, 1105 AZ, Amsterdam, The Netherlands; 3 Department of Neuroscience, Erasmus MC, 3000 CA, Rotterdam, The Netherlands; 4 Netherlands Institute for Neuroscience, Royal Dutch Academy of Arts & Sciences, 1105 BA, Amsterdam, The Netherlands; 5 Leiden Institute of Chemistry, Leiden University, 2300 RA, Leiden, The Netherlands; 6 Department of Internal Medicine, Hospital of Bregenz, 6900, Bregenz, Austria; University Hospital S. Maria della Misericordia, Udine, ITALY

## Abstract

The enzyme glucocerebrosidase (GBA) hydrolyses glucosylceramide (GlcCer) in lysosomes. Markedly reduced GBA activity is associated with severe manifestations of Gaucher disease including neurological involvement. Mutations in the *GBA* gene have recently also been identified as major genetic risk factor for Parkinsonism. Disturbed metabolism of GlcCer may therefore play a role in neuropathology. Besides lysosomal GBA, cells also contain a non-lysosomal glucosylceramidase (GBA2). Given that the two β-glucosidases share substrates, we speculated that over-activity of GBA2 during severe GBA impairment might influence neuropathology. This hypothesis was studied in Niemann-Pick type C (*Npc1* ^-/-^) mice showing secondary deficiency in GBA in various tissues. Here we report that GBA2 activity is indeed increased in the brain of *Npc1* ^-/-^ mice. We found that GBA2 is particularly abundant in Purkinje cells (PCs), one of the most affected neuronal populations in NPC disease. Inhibiting GBA2 in *Npc1* ^-/-^ mice with a brain-permeable low nanomolar inhibitor significantly improved motor coordination and extended lifespan in the absence of correction in cholesterol and ganglioside abnormalities. This trend was recapitulated, although not to full extent, by introducing a genetic loss of GBA2 in *Npc1* ^-/-^ mice. Our findings point to GBA2 activity as therapeutic target in NPC.

## Introduction

Glucocerebrosidase (GBA) is a membrane-associated enzyme that hydrolyses the β-glucosyl linkage of glucosylceramide (GlcCer) in lysosomes [1]. Inherited deficiency of GBA due to mutations in the *GBA* gene causes Gaucher disease (GD). This lysosomal storage disorder is remarkably heterogeneous in onset, progression and nature of symptoms. In severely affected GD patients with marked reduction in GBA activity neurological symptoms develop. Of interest, mutations in the *GBA* gene have recently been associated with development of Parkinson disease (PD) and other Lewy bodies disorders [2]. Mutations in the *SCARB2* gene encoding the membrane protein LIMP2, which mediates the transport of GBA to lysosomes, have also been reported to constitute a risk factor for PD [3].

Cells do not rely only on GBA to degrade GlcCer. Another glucosylceramidase, the non-lysosomal GBA2, can also hydrolyze GlcCer to ceramide and glucose in the cytosol [4]. GBA2 is a non-integral membrane-associated protein located at the endoplasmic reticulum and Golgi [5,6]. Several studies have pointed towards the existence of a compensatory mechanism between GBA and GBA2 [7–9]. For example, increased GBA2 activity has been recently reported in brain of Gaucher mice and in leukocytes of Gaucher patients [7]. We earlier speculated that GBA2 might be involved in GD etiology [9,10]. Very recently, Mistry and colleagues demonstrated that *Gba2* gene deletion rescues the visceral, hematologic, and skeletal phenotype in a non-neuronopathic GD mouse model with impaired GBA activity in the white blood cell lineage [8]. Unfortunately, this animal model is not suitable to study the impact of GBA2 on neurological manifestations. An alternative approach to study this is offered by Niemann-Pick type C (NPC) disease. It is well documented that in tissues and cultured fibroblasts of NPC patients, GBA activity is secondarily reduced [11–13]. NPC is a neurodegenerative lysosomal storage disease caused by loss-of-function mutations in either the *Npc1* or *Npc2* genes, encoding proteins essential for the export of cholesterol from lysosomes [14]. NPC patients develop ataxic gait, motor dysfunction and seizures [15]. Next to accumulation of cholesterol, glycosphingolipids (GSLs), particularly gangliosides, accumulate in the brain of NPC patients [15]. A mouse model for NPC, *Npc1* ^-/-^ mice, is available that offers a phenocopy of the disease in man, including the characteristic neurological manifestations such as a striking defect in motor coordination [16]. We therefore examined whether compensation between GBA and GBA2 occurs in *Npc1* ^-/-^ mice, and if so, whether GBA2 activity mediates neuropathology in the animals and is amenable to pharmacological correction with specific inhibitors. Here we report the outcome of our investigation pointing to GBA2 activity as a potential therapeutic target in NPC.

## Material and Methods

### Animal studies

*Npc1* ^*-/-*^ mice, along with wild-type littermates (*Npc1* ^+/+^), were generated by crossing *Npc1* ^+/-^ males and females in-house. The heterozygous BALB/c Nctr-*Npc1* ^*m1N*^/J mice (stock number 003092) were obtained from The Jackson Laboratory (Bar Harbor, USA). Mouse pups were genotyped according to published protocols [16]. Mice (± 3 weeks old) received the rodent AM-II diet (Arie Blok Diervoeders, Woerden, The Netherlands). For the treatment groups, n = 6 per group unless stated differently, the diet was supplemented with 6 or 30 mg MZ-21 or MZ-31 per kilogram AM-II diet to obtain the doses of 1 and 5 mg per kg body weight per day, respectively.

The *Gba2* ^*-/-*^ mice (C57Bl/6-129S6/SvEv mixed background) were generated as previously described [17]. *Npc1* ^-/-^/*Gba2* ^-/-^ mice were generated by crossing single heterozygous *Npc1* ^+/-^ and single knockout *Gba2* ^-/-^ mice to produce compound heterozygotes. These mice were crossed to generate double-knockout mice and other combinations. All the mice generated have 50% 129S6/SvEv-C57Bl/6 and 50% BALB/c background. Body weight, physical activity and motor coordination of the mice were monitored (n = 5–6 per group).

Objective criterion for final euthanasia of *Npc1* ^-/-^ mice, along with WT controls, was weight loss greater than 30% of peak weight accompanied by hunched posture and reduced activity. We also checked that at this stage the animals were no longer able to remain upright due to trembling/shaking. Mice body weight was recorded on a Tuesday-Friday schedule. Once a week a movie of 1 min was made to record the clinical symptoms. There were no unexpected deaths of animals used in the study. When the mice presented reduced activity hydration gel packs and wet food were placed in the cage to facilitate access to fluids and food. Animals were anesthetized and sacrificed by cervical dislocation (see next section for details).

The mice were housed at the Institute Animal Core Facility in a temperature- and humidity-controlled room with a 12-h light/dark cycle and given free access to food and water ad libitum. All animal protocols were approved by the Institutional Animal Welfare Committee of the Academic Medical Centre Amsterdam in the Netherlands.

### Tissue processing and immunohistochemistry

Animals were first anesthetized with a dose of Hypnorm (0.315 mg/mL fenyl citrate and 10 mg/mL fluanisone) and Dormicum (5 mg/mL midazolam) according to their weight. The given dose was 80μL/10g body weight. Blood was collected by a heart puncture followed by cervical dislocation. Brains were dissected, rinsed with phosphate-buffered saline (PBS) and divided by median section. The left half was fixed in phosphate-buffered formalin and further used for histology. The right half of non-perfused brain was snap frozen in liquid N_2_ and stored at -80°C for biochemistry. Later, homogenates from the frozen material were made in 25 mM potassium phosphate buffer, pH 6.5, supplemented with 0.1% (v/v) Triton X-100 and protease inhibitors.

Formalin-fixed, paraffin-embedded tissue was sectioned at 4-μm thickness, dried overnight at 37°C, deparaffinized in xylene and rehydrated in graded alcohol. Endogenous peroxidase activity was blocked by incubation for 10 min in methanol containing 0.3% H_2_O_2_. Sections were rehydrated and slides were heated in 0.04 M citrate, 0.12 M phosphate, pH 5.8, for 10 min at 121°C. After washing, the sections were incubated with primary antibody being either polyclonal rabbit IgG anti-mouse GBA2 (#25, 1:500; produced by Eurogentec [Maastricht, The Netherlands] with the peptides CGSPEDSGPQDEPSY and GRYYNYDSSSHPQSR), polyclonal rabbit IgG anti-calbindin D-28K (PC253L, 1:1,000; Calbiochem, San Diego, CA, USA), polyclonal rabbit IgG anti-GBA (C-terminal, G4171, 1:1,000, Sigma-Aldrich, St Louis, MO, USA), polyclonal rabbit IgG anti-GFAP (Z0334, 1:2,000; Dako, Glostrup, Denmark), or monoclonal mouse IgG1 anti-neurofilament (2F11, 1:250; Thermo Fisher Scientific, Rockford, IL, USA) in Antibody Diluent (ImmunoLogic, Klinipath, Duiven, The Netherlands) for 16 hours at 4°C, washed, and incubated with secondary antibody being either poly-HRP goat anti-rabbit IgG (BrightVision, ImmunoLogic), or HRP-conjugated rabbit IgG anti-rat IgG (P0450, 1:3,000; DAKO) followed by poly-HRP goat anti-rabbit IgG, or HRP-conjugated goat anti-mouse IgG1 (1070–05; SouthernBiotech, Birmingham, AL, USA). HRP activity was detected using 3,3’-diaminobenzidine as substrate. Sections were counter-stained with hematoxylin and mounted with pertex.

In addition, after blocking endogenous peroxidase activity and epitope retrieval as indicated above, sections were subsequently incubated with 5% (v/v) normal goat serum, rabbit anti-GBA, and intestinal alkaline phosphatase-conjugated goat anti-rabbit IgG (BrightVision, ImmunoLogic). Intestinal AP activity was detected using VectorBlue substrate (SK-5300; Vector Laboratories, Burlingame, CA, USA) in presence of 0.2 mM levamisole to inhibit endogenous non-intestinal alkaline phosphatase activity. Next, epitope retrieval (10 min, 121°C) in citrate buffer (pH 5.8) was applied to remove all antibodies [18]. Subsequently, sections were incubated with normal goat serum, rabbit anti-GBA2, and HRP-conjugated goat anti-rabbit IgG. HRP activity was detected using ImmPACT AMEC Red substrate (SK-4285; Vector Laboratories). Sections were counterstained with methyl green and mounted with VectaMount (Vector Laboratories). Analysis was performed using brightfield microscopy (Leica DM5000B) with an HC PLAN APO 20x/0.70 objective. Multispectral data sets were acquired using a Nuance imaging system (Perkin Elmer, Hopkinton, MA, USA) from 420 to 720 nm at intervals of 10 nm. Spectral libraries for each chromogen were obtained from single-stained sections and were used to unmix the triple staining patterns. Nuance 3.0.2 software was used to construct composite images applying color universal design. Images were color inverted using Adobe Photoshop to enhance visibility for color-blind individuals.

### *In vivo* labeling of GBA2 in rat brain

Wistar rats (300 g) (Charles River Laboratories, Wilmington, MA, USA) were implanted with intracerebroventricular (ICV) cannulas using the coordinates: AP -0.9, L +2.0 and V -3.4. After a recovery period conduritol-β-epoxide (1 μM) was infused at a rate of 1 μL per minute for 10 min. After one hour, ABP 1 (10 nM) [19] was infused at a rate of 1 μL per minute for 10 min. After 4 h the animals were sacrificed and the brains were frozen. Thirty-μm-thick cryostat sections were prepared and extensively washed. After overnight incubation with rabbit anti-calbindin D-28K antibody, the secondary Alexa-488-conjugated donkey anti-rabbit IgG antibody (R37116, Invitrogen, Carlsbad, CA, USA) was added and incubated for 1 h at RT. The sections were mounted with Vectashield (Vector Laboratories, Burlingame, CA, USA) containing DAPI and fluorescence was imaged using confocal laser scanning microscopy (Leica TCS SP5, Leica Microsystems, Wetzlar, Germany).

### Enzyme activity assays

All 4-methylumbelliferyl (4-MU) substrates used were obtained from Sigma (Sigma-Aldrich, Germany). GBA activity was assayed as previously described [20]. GBA2 activity was assayed in McIlvaine buffer, pH 5.8, with 0.1% (w/v) BSA, after pre-incubation with 100 nM MDW933 [20] for 30 min at 37°C. Β-hexosaminidase activity was measured with 1.97 mM 4-MU-N-acety-β-D-glucosaminide in 150 mM citrate-Na_2_HPO_4_ (pH 4.0) buffer. Β-glucuronidase activity was measured with 2 mM 4-MU-β-D-glucuronide in 100 mM sodium acetate (pH 4.8) buffer.

### Gel electrophoresis and fluorescence scanning

Electrophoresis in sodium dodecylsulfate (SDS) containing 7.5% polyacrylamide gels was performed as earlier described [19].

### Western Blot

Equal amounts of protein (50 μg) were subjected to electrophoresis on 7.5% SDS-polyacrylamide gels and then transferred to nitrocellulose membranes (Whatman, Dassel, Germany) using an electroblotting apparatus (Bio-Rad Laboratories, Hercules, CA, USA). The blots were blocked in 5% (w/v) nonfat dried milk in TBST buffer (10 mM Tris-HCl [pH 8.0], 150 mM NaCl, 0.05% [v/v] Tween-20) and probed with anti-GBA2 (1:1,000), anti-GBA (1:1,000) or anti-tubulin (clone DM1A, ascites fluid, 1:10,000, Sigma-Aldrich, St Louis, MO, USA) antibody diluted in blocking buffer, overnight at 4°C. After washing, the membranes were incubated with secondary antibody (anti-rabbit/mouse IgG IRDye 800CW [Westburg, Leusden, The Netherlands]) diluted 1:10,000 in blocking buffer, for 1 h at RT. Blots were scanned on an Odyssey image scanner (GE Healthcare, Munich, Germany).

### Histochemistry and Purkinje cell (PC) quantification

Tissue sections were stained with hematoxylin and eosin (HE). Parasagittal sections of cerebellum were scanned using an Olympus BX61VS microscope with UPlanSApo 20x/0.75 objective and TIFF images were acquired using the Olympus dotSlide system (Olympus, Tokyo, Japan). All those PCs were counted in which a nucleus with a nucleolus was observed. The PC layer was traced and length of the trace was measured using ImageProPlus 7.0 software (Media Cybernetics, Rockville, MD, USA). The number of PCs per unit of PC layer was quantified for the anterior, central, posterior, and flocculonodular transversal zones of the cerebellum [21].

### Erasmus ladder

We tested motor coordination of untreated and iminosugar treated (5 mg/kg.bw/day of MZ-21) *Npc1* ^+/+^, *Npc1* ^+/-^ and *Npc1* ^-/-^ mice using the Erasmus Ladder. Details on the device and its software have been published before [22–24]. Both the recording and stimulating parts of the setup are controlled by software written in LabView (National Instruments, Austin, TX, USA) operating at a fixed cycle of 2 ms. All data collected are stored in a relational database (MySQL, Oracle, Redwood Shores, CA, USA) and subsequently processed and analyzed off-line using custom-written software in LabView and Python (Python Software Foundation, Beaverton, OR, USA) as well as SPSS (IBM Corporation, Armonk, NY, USA). For the current study we followed a modified version of a previously developed protocol [24]. In principle, if health status permitted, each mouse had to perform 1 daily session during 5 days at the age of 6 weeks and one daily session during 2 days at the age of 9 and 10 weeks. The sessions for the 6 weeks-old stage included in consecutive order 3 non-perturbed sessions so as to adjust to the apparatus, 1 fix-obstacle session during which rung number 19 remained always elevated by 18 mm (i.e. 12 mm above the walking path), and 1 paired session in which the unconditioned stimulus (i.e. elevated rung) was provided at a random location on the right side 200 ms following the onset of the conditioned stimulus (i.e. a tone), which in turn depended on the predicted trajectory of the mouse (for details see [22]). The sessions for the 9 and 10 weeks-old stages included 1 non-perturbed session followed by 1 fix-obstacle session. During all sessions mice had to walk back and forth between the 2 shelter boxes, and during all sessions, which always included 45 trials, we took step time as the main outcome parameter, which was defined as the time (in ms) that elapses between the onsets of two consecutive touches on the rungs.

### Cholesterol measurement

Total cholesterol levels in brain homogenates were determined after lipid extraction according to Folch [25] using a colorimetric enzymatic kit (Biolabo, Maizy, France).

### Ganglioside quantification

Ganglioside composition was determined as previously described [26]. Asialo-ganglioside GM1 from bovine brain (Sigma, St Louis, MO, USA) was used as an internal standard.

### (Glyco)sphingolipid quantification

Levels of ceramide and of the isomers glucosylceramide and galactosylceramide were determined in homogenates of total brain after lipid extraction by modified Bligh and Dyer method [27]. Neutral glycosphingolipids were separated from neutral lipids (ceramides and phospholipids) by Solid Phase Extraction (SPE) using a Backergrond Silica Gel (SiOH) SPE column (1 mL, 100 mg per column). After deacylation with sphingolipid ceramide *N*-deacylase (SCDase, Takara Bio Inc., Japan), glucosylceramide-borne glucosylsphingosine was digested with recombinant GBA (imiglucerase, Genzyme) yielding freed sphingosine. Sphingoid bases and lyso-glycosphingolipids were derivatized with *O*-phthaldialdehyde and analyzed by HPLC as described before [28].

### Glucosylsphingosine quantification

Glucosylsphingosine analysis was performed according to a previously described protocol [29].

### Statistical analysis

Values presented in figures concerning the *Npc1* ^-/-^ mice represent mean ± s.e.m.. Statistics were performed using the nonparametric Kruskal-Wallis test followed by a *post hoc* Dunn’s multiple comparison test using GraphPad Prism 5 (Graph Pad Software, Inc., San Diego, USA): * *P* < 0.05; ** *P* < 0.01; *** *P* < 0.001.

## Results

### GBA and GBA2 in wild-type and *Npc1* ^-/-^ mouse brain

**Fig 1:**
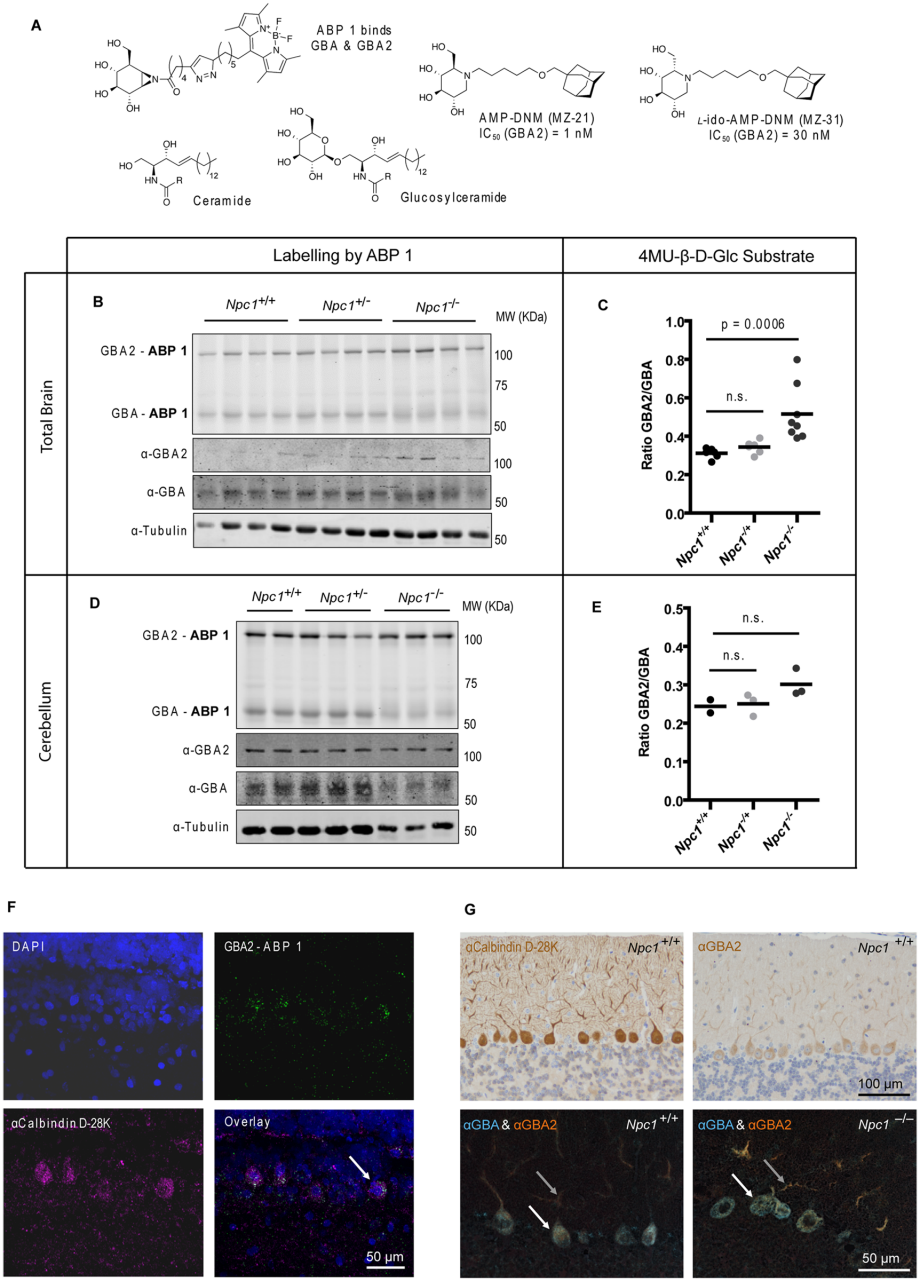
GBA and GBA2 in brain of *Npc1* ^+/+^ and *Npc1* ^-/-^ mice. (A) Chemical structures of ceramide (Cer), glucosylceramide (GlcCer), the ABP 1 specific for GBA and GBA2, and the inhibitors AMP-DNM (MZ-21) and *l*-ido-AMP-DNM (MZ-31) with respective IC_50_ values. (B) Fluorescent labelling of GBA and GBA2 with ABP 1 and Western blotting with anti-GBA2, anti-GBA and anti-tubulin antibodies in brain homogenates of 85-day-old *Npc1* ^*+/+*^, *Npc1* ^*+/-*^ and *Npc1* ^*-/-*^ mice. (C) Ratio of GBA2 and GBA enzymatic activities (assayed with 4MU-β-D-Glc substrate) in brain homogenates of 85-day-old *Npc1* ^*+/+*^, *Npc1* ^*+/-*^ and *Npc1* ^-/-^ mice. (D) Fluorescent labelling of GBA and GBA2 with ABP 1 and Western blotting with anti-GBA2, anti-GBA and anti-tubulin antibodies in homogenates of dissected cerebella of 75-day-old *Npc1* ^*+/+*^, *Npc1* ^*+/-*^ and *Npc1* ^*-/-*^ mice. (E) Ratio of GBA2 and GBA enzymatic activities (assayed with 4MU-β-D-Glc substrate) in cerebellar homogenates of 75-day-old *Npc1* ^*+/+*^, *Npc1* ^*+/-*^ and *Npc1* ^-/-^ mice. (F) Fluorescent labelling of GBA2 with ABP 1 (top-right) in wild-type rat cerebellum by ICV injection. GBA was pre-blocked with conduritol-β-epoxide. Sections were also labelled with DAPI (top-left) and immunostained with anti-calbindin D-28K antibody (bottom-left). The arrow indicates a double-labelled cell. Scale bar = 50 μm. (G) Single immunostaining with anti-calbindin D-28K and anti-GBA2 antibodies (top) and double immunostaining with anti-GBA and anti-GBA2 antibodies (bottom) of cerebellar sagittal sections of 85-day-old *Npc1* ^+/+^ and *Npc1* ^-/-^ mice. The arrows indicate PC cell body (white) and dendrites (grey). Scale bars = 100 μm (top) and 50 μm (bottom).

The enzymes GBA and GBA2 in brain of end-stage *Npc1* ^-/-^ mice were examined employing a recently developed activity-based probe to visualize these two β-glucosidases [19]. The activity-based probe (ABP 1), a fluorescent aziridine phellitol-type suicide inhibitor (Fig 1A), labels with high specificity and sensitivity through covalent binding to the catalytic nucleophile residue in active GBA (half maximal inhibitory concentration (IC_50_) = 1.15 nM) and GBA2 (IC_50_ = 0.15 nM) enzyme molecules [19]. The two fluorescently-labeled enzymes can each be quantitatively visualized following separation by SDS-PAGE electrophoresis.

While relative content of active GBA as assessed by ABP 1 labelling was not markedly reduced (Fig 1B), active GBA2 was nevertheless found to be increased in lysates of brain of 85-day-old *Npc1* ^-/-^ mice (Fig 1B, see S1 Fig for band intensity quantification). Accordingly, measurement of the activity of the two β-glucosidases with the 4-methylumbelliferyl-β-D-glucopyranoside (4MU-β-D-Glc) fluorogenic substrate revealed a significant increase (*P* = 0.0006) of the GBA2/GBA ratio in *Npc1* ^-/-^ mice brain lysates (Fig 1C, see S1 Fig for individual activities). Increased GBA2 in brain of *Npc1* ^-/-^ mice was also observed at the protein level by Western blot analysis with anti-GBA2 antibody (Fig 1B and S1 Fig). The cerebellum is one of the brain areas most affected by neuropathology in NPC patients and *Npc1* ^-/-^ mice. In dissected cerebella of 75-day-old *Npc1* ^-/-^ mice changes in relative amount of active GBA and GBA2 as detected by ABP 1 labelling were less clear than in total brain (Fig 1D and S1 Fig) and the ratio of GBA2/GBA activity assayed with artificial substrate was slightly (but not significantly) increased (Fig 1E and S1 Fig) compared to those in control wild-type (wt) mice. A similar increase in the ratio was observed for the other brain areas analyzed (S2 Fig).

Next, we performed intracerebroventricular (ICV) infusion of ABP 1 into living rats. ABP 1 localized mainly to calbindin D-28K positive Purkinje cells (PCs) in the cerebellum (Fig 1F). GBA labeling was blocked by pre-infusion with conduritol-β-epoxide. These findings strongly suggest that PCs express active GBA2 *in vivo* (Herrera Moro *et al*. to be published in detail). Using immunostaining on mouse cerebellum we found that GBA2 is particularly abundant in these sole output neurons of the cerebellar cortex that are critical for motor coordination (Fig 1G) [30]. Double immunostaining revealed GBA2 to be present in the PC cell body as well as the dendrites, whereas GBA is largely restricted to the PC cell body in a punctate distribution pattern in agreement with lysosomal localization (Fig 1G).

We then studied the natural history of neuropathology in *Npc1* ^-/-^ mice with special attention to GBA and GBA2. As reported earlier by others [31], progressive loss of PCs started in the anterior cerebellum, as reflected by decreased staining for calbindin D-28K. Concomitantly, astrogliosis (α-GFAP) increased in aging *Npc1* ^-/-^ mice. Surviving *Npc1* ^-/-^ PCs in the central and more posterior cerebellum continued to express GBA and GBA2 (S3 Fig). Enlarged vesicular structures in *Npc1* ^-/-^ PCs at day 80 did not stain for either GBA or GBA2, whereas the remainder of the PC cell body as well as the disorganized dendrites often co-stained for both GBA and GBA2 (Fig 1G). Both GBA and GBA2 accumulated in axonal swellings that were observed at days 60 and 80 in *Npc1* ^-/-^ cerebella (S3 Fig).

### Impact of genetic deficiency of Gba2 on *Npc1* ^-/-^ mice

**Fig 2:**
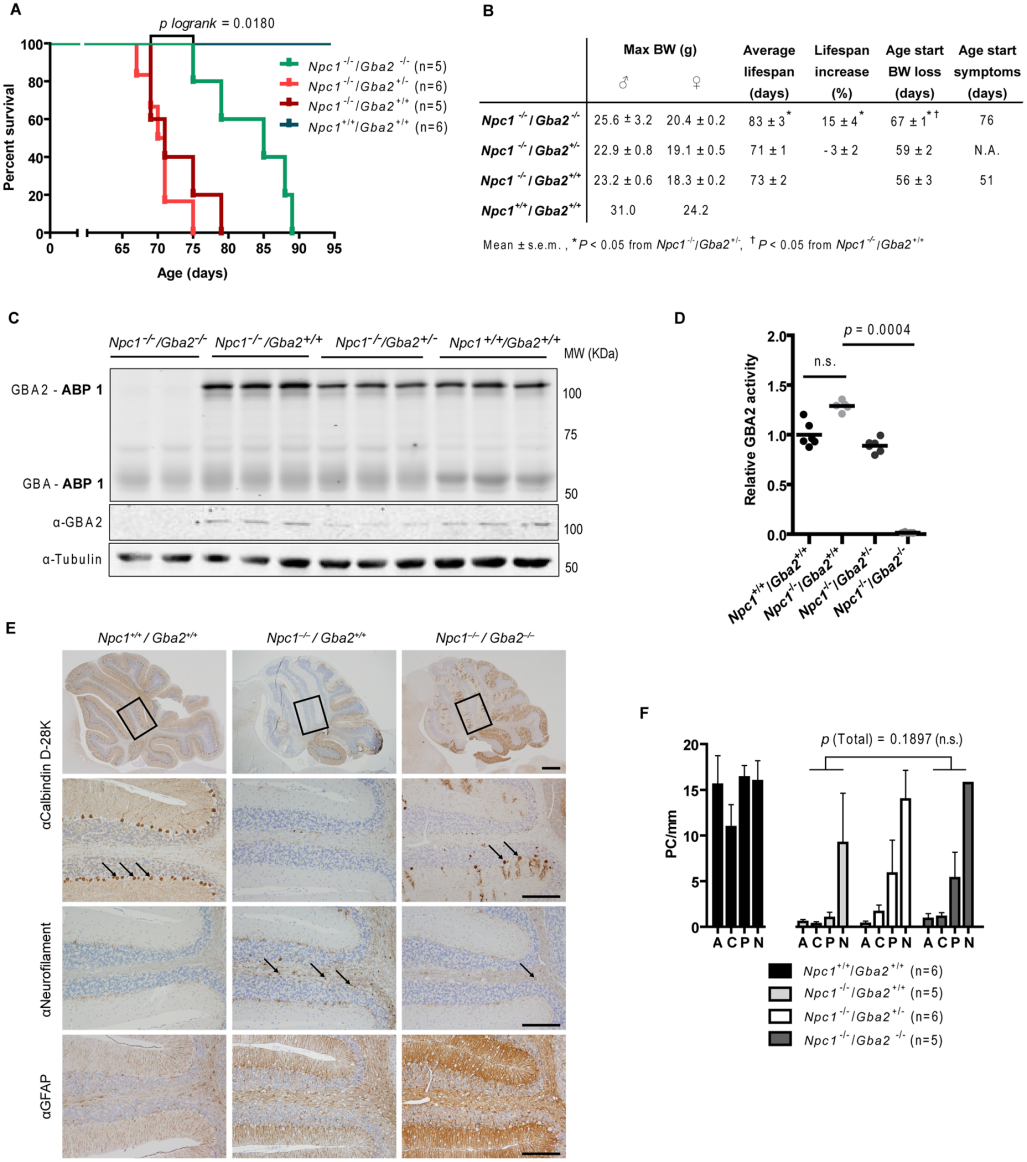
Crossing of *Gba2* ^*-/-*^ (C57BL/6-129S6/SvEv) mice with *Npc1* ^*-/-*^ (BALB/c) mice. (A) Survival Kaplan-Meier plot of transgenic mice resulting from the crossing of GBA2 deficiency into the *Npc1* ^*-/-*^ background (*P logrank* = 0.0180 from *Npc1* ^*-/-*^/*Gba2* ^+/+^). (B) Maximal bodyweight, average lifespan, percentage lifespan increase and age at which bodyweight loss and neurological symptoms are patent in crossed animals. (C) Fluorescent labelling of GBA and GBA2 with ABP 1 (100 nM) and Western blotting with anti-GBA2 and anti-tubulin antibodies in brain homogenates of end-stage transgenic male mice. (D) GBA2 activity (assayed with 4MU-β-D-Glc substrate) in brain homogenates of end-stage transgenic mice relative to *Npc1* ^*+/+*^/*Gba2* ^+/+^ (wt) values. (E) Sagittal cerebellar sections of end-stage transgenic mice immunostainned with anti-calbindin D-28K, -neurofilament and -GFAP antibodies. The arrows indicate calbindin D-28K positive cells (2^nd^ panel from top) and swollen axons positive for neurofilament (3^rd^ panel). Scale bar = 500 μm (top-panel) and 200 μm (other panels). (F) PC count in the anterior (A), central (C), posterior (P) and flocculonodular (N) transversal zones of the cerebellum of end-stage transgenic mice. Data (*n* = 5–6 per group, mean ± s.e.m.) were analyzed using the Kruskal-Wallis test with *post hoc* Dunn’s multiple comparison test.

**Fig 3:**
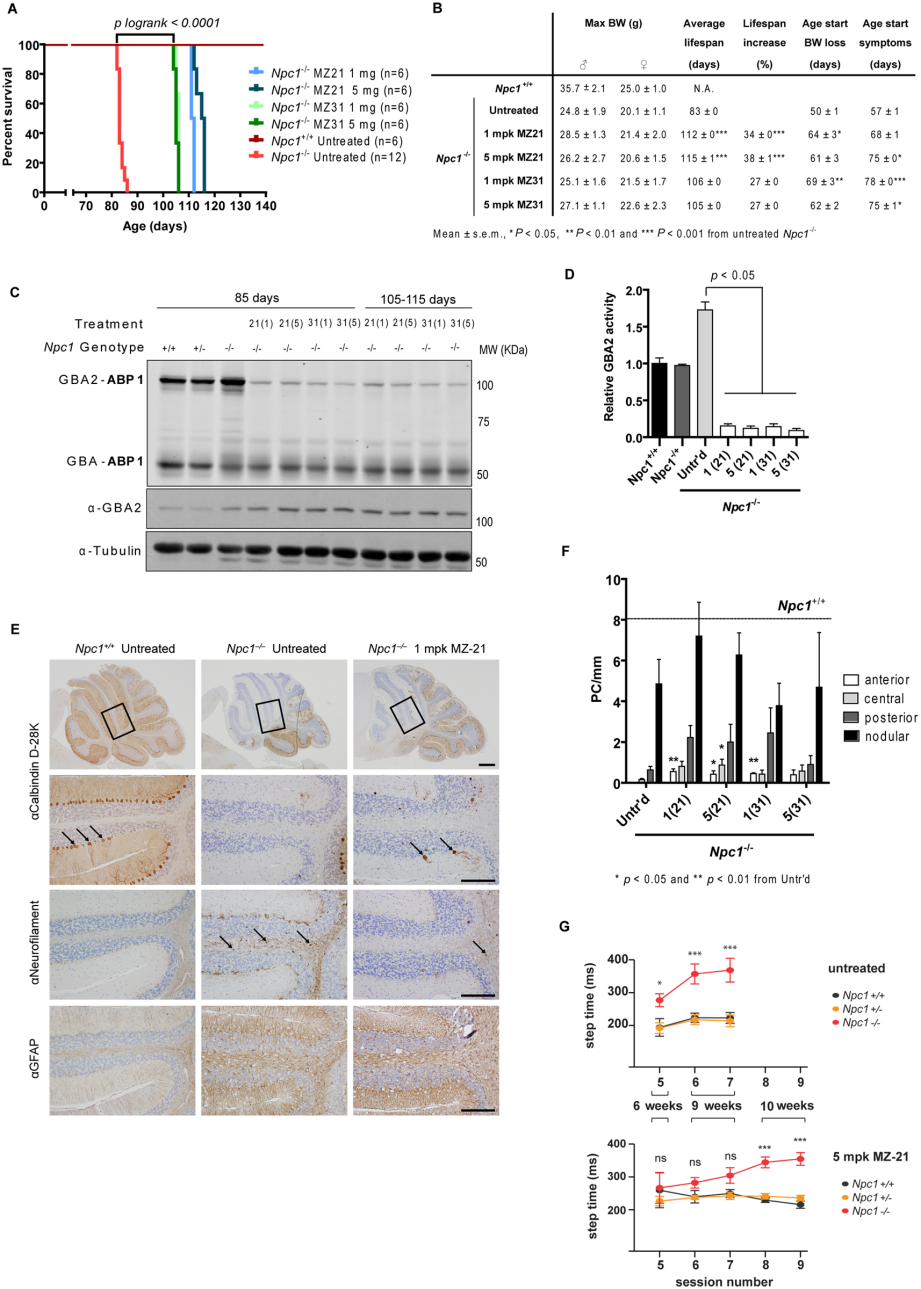
Pharmacological inhibition of GBA2 in *Npc1* ^-/-^ (BALB/c) mice. (A) Kaplan-Meier survival plots of *Npc1* ^-/-^ mice treated with 1 and 5 mg/kg.bw/day (mpk) of MZ-21 and MZ-31 (*P logrank* < 0.0001 from untreated *Npc1* ^-/-^ mice). (B) Maximal bodyweight, average lifespan, percentage lifespan increase and age at which bodyweight loss and neurological symptoms are patent in *Npc1* ^*+/+*^ and *Npc1* ^-/-^ treated and untreated mice. (C) Fluorescent labelling of GBA and GBA2 with ABP 1 and immunostaining with anti-GBA2 and -tubulin antibodies in brain homogenates of *Npc1* ^*+/+*^, *Npc1* ^*+/-*^ and *Npc1* ^-/-^ treated and untreated 85- and 110-day-old mice. (D) GBA2 activity (assayed with 4MU-β-D-Glc substrate) in brain homogenates of *Npc1* ^*+/+*^, *Npc1* ^*+/-*^ and *Npc1* ^-/-^ treated and untreated 85-day-old mice relative to *Npc1* ^*+/+*^ (wt) values. (E) Sagittal cerebellar sections of 85-days-old *Npc1* ^*+/+*^ and *Npc1* ^*-/-*^ treated and untreated immunostainned with antibodies against calbindin D-28K, neurofilament and GFAP. The arrows indicate calbindin D-28K positive cells (2^nd^ panel from top) and swollen axons positive for neurofilament (3^rd^ panel). Scale bar = 500 μm (top-panel) and 200 μm (other panels). (F) PC count in the anterior, central, posterior and flocculonodular areas of the cerebellum of *Npc1* ^-/-^ treated and untreated 85-day-old mice. The line indicates *Npc1* ^+/+^ levels. (G) Motor coordination of untreated and treated *Npc1* ^-/-^ mice measured in the Erasmus Ladder. Step time (in ms) during perturbed sessions of *Npc1* ^*+/+*^, *Npc1* ^*+/-*^ *and Npc1* ^-/-^ untreated (top) and treated (bottom) mice at six (session 5), nine (sessions 6 & 7) and ten (sessions 8 & 9) weeks of age. Data (*n* = 4–12 per group, mean ± s.e.m.) were analyzed using the Kruskal-Wallis test with *post hoc* Dunn’s multiple comparison test: * *P* < 0.05; ** *P* < 0.01; *** *P* < 0.001.

Since GBA2 activity was found to be relatively increased in *Npc1* ^-/-^ mice (Fig 1B and 1C), we tested the consequence of introducing a genetic *Gba2* loss in these animals. For this we crossed *Gba2*-deficient mice (C57BL/6-129S6/SvEv) without an overt phenotype [17] with *Npc1* ^-/-^ mice (BALB/c) to generate combined *Npc1* ^-/-^/*Gba2* ^-/-^. The genetic background of *Npc1* ^-/-^ mice is reported to influence disease severity, being least severe in BALB/c animals [32]. This finding was recapitulated: the average lifespan of *Npc1* ^*-/-*^ mice with mixed background being 73 days (Fig 2A and 2B) compared to 83 days for animals with BALB/c background (see also Fig 3A and 3B). In brains of *Npc1* ^-/-^/*Gba2* ^+/+^ mice with mixed background total GBA2 activity and protein were elevated at end-stage (Fig 2C and 2D, S4 Fig), similar to that seen for animals with the BALB/c background (Fig 1B and 1C).

Body weight, physical activity and motor coordination of mice with various combinations of deficiencies in *Npc1* and *Gba2* were monitored. Mice were euthanized when weight loss exceeded 30% of peak weight and the animals presented hunched posture, reduced activity and were no longer able to remain upright due to trembling/shaking. As shown in the Kaplan-Meier survival curves (Fig 2A), combined loss of *Gba2* prolonged lifespan significantly in *Npc1* ^*-/-*^ mice (*P logrank* = 0.0180 from *Npc1* ^-/-^/*Gba2* ^+/+^) and increased maximal bodyweight (Fig 2B) compared to animals wild-type or heterozygous for this enzyme. Neurological symptoms—defective coordination and shaking (ataxia)–were delayed by 15 days in double-knockout mice (Fig 2B). Of note, *Gba2* deficiency did significantly delay, but not fully prevent complications. PC number appeared higher in the transversal cerebellar zones of 83-day-old *Npc1* ^-/-^/*Gba2* ^-/-^ mice compared to 73-day-old *Npc1* ^-/-^/*Gba2* ^+/+^ animals (Fig 2E and 2F), nevertheless this difference did not reach statistical significance (*P* = 0.1897, Fig 2F). Cerebellar calbindin D-28K expression was more widespread and neurofilament containing axonal swelling was less abundant in double-knockout mice compared to *Npc1* ^-/-^/*Gba2* ^+/+^ controls (Fig 2E). In contrast, astrogliosis was not diminished by the genetic deficiency of *Gba2*, as evidenced by abundant GFAP staining (Fig 2E).

### Impact of pharmacological inhibition of GBA2 on *Npc1* ^-/-^ mice

Having established that genetic loss of *Gba2* ameliorates neuropathology in *Npc1* ^-/-^ mice, we investigated whether such beneficial effect can also be obtained by pharmacological inhibition of GBA2 activity. We have previously designed very potent inhibitors of GBA2, the hydrophobic aza-sugars AMP-DNM (MZ-21) and ido-AMP-DNM (MZ-31) (Fig 1A) [33]. Both aza-sugars enter the brain well and inhibit GBA2 at low nanomolar concentration [34].

*Npc1* ^*-/-*^ mice (BALB/c background) were fed a diet calculated to deliver 1 or 5 mg/kg.bw/day of MZ-21 or MZ-31. Again, iminosugar-mediated reduction of GBA2 activity increased lifespan by 20–30 days (Fig 3A and 3B) and significantly delayed onset of weight loss and clinical manifestations of neurodegeneration (Fig 3B). We found that in iminosugar-treated animals GBA2 enzyme activity (Fig 3C and 3D), but not GBA2 protein (Fig 3C and S5 Fig), was completely abrogated with either iminosugar at both doses. Additionally, the number of PCs in the cerebellum of 85-day-old iminosugar-treated *Npc1* ^*-/-*^ mice was significantly higher (*P* < 0.05, Fig 3F) than in age-matched untreated animals as assessed by PC counts in HE stainings (Fig 3F) and calbindin D-28K immunostaining (Fig 3E). Further consistent with improved clinical status, axonal swelling in *Npc1* ^*-/-*^ mice as indicated by neurofilament immunostaining was less extensive following iminosugar treatment (Fig 3E). Extent of GFAP-positive astrogliosis however was not affected (Fig 3E). Also staining for activated microglia and macrophages, using Iba-1 and F4/80, were largely similar in *Npc1* ^*-/-*^ mice with and without iminosugar treatment (S6 Fig).

To evaluate the effect of pharmacological inhibition of GBA2 on motor coordination we tested iminosugar-treated and untreated *Npc1* ^+/+^, *Npc1* ^+/-^ and *Npc1* ^-/-^ mice on the Erasmus Ladder [24]. Untreated *Npc1* ^*-/-*^ mice needed significantly longer time to make a single step than *Npc1* ^+/+^ controls at the ages of 6 (paired session, *P* = 0.0022) and 9 weeks (non-perturbed session, *P =* 0.001; fix-obstacle session, *P =* 0.002) (Fig 3G). Remarkably, these differences were not observed for the iminosugar-treated animals during the fix-obstacle session (*P =* 0.060) (Fig 3G). Only at 10 weeks of age did iminosugar-treated *Npc1* ^*-/-*^ mice need significantly longer time than controls to make a single step during the non-perturbed (*P* < 0.001) and the fix-obstacle (*P* < 0.001) sessions (Fig 3G). Thus, as time progressed iminosugar-treated *Npc1* ^*-/-*^ mice also developed impaired motor coordination, but the onset of the impairments was delayed by about four weeks.

### Brain lipids and lysosomes in iminosugar treated *Npc1* ^-/-^ mice

Given the primary role of cholesterol and ganglioside accumulation in lysosomes in NPC, we next analyzed the impact of iminosugar treatment of *Npc1* ^*-/-*^ mice on brain lipid composition and lysosomes. No significant differences between total brain cholesterol and ceramide levels of age-matched (85-day-old) *Npc1* ^*+/+*^, treated or untreated *Npc1* ^*-/-*^ mice were detected (Table 1). Although not statistically significant, ceramide levels showed the trend to be elevated in total brain of *Npc1* ^*-/-*^ mice compared to *Npc1* ^+/+^ controls and tended to normalize with most iminosugar treatment conditions (Table 1). In contrast, GlcCer and the gangliosides GM3 and GM2 were increased in the brain of *Npc1* ^*-/-*^ untreated mice and these levels were not normalized by MZ-21 or MZ-31 treatment (Table 1). Rather, GlcCer was more than 10-fold increased by iminosugar treatment (Table 1). The de-acylated form of GlcCer, glucosylsphingosine (GlcSph), was also elevated in untreated animals and further increased by the treatment (Table 1). Galactosylceramide (GalCer), the main component neuronal myelin sheaths, levels were reduced by half in the brain of *Npc1* ^-/-^ mice, confirming that the profound CNS demyelination in these animals was not corrected by the treatment.

**Table 1:** Inhibition of GBA2 activity does not correct lysosomal defects.

			Cholesterol (μmol/g ww)	Glycosphingolipids (nmol/g ww)						Activity (% *wt*)	
Genotype	Age (d)	Treatment		GalCer (% *wt*)	GlcCer (% *wt*)	Cer (% *wt*)	GlcSph	GM3	GM2	β-hexo	β-glucuro
***Npc1*** ^***+/+***^	**85**		29.8 ± 0.3	100 ± 2	100 ± 1	100 ± 7	0.2 ± 0.0	12 ± 1	12 ± 4	100 ± 13	100 ± 14
***Npc1*** ^***+/-***^	**85**		33.2 ± 0.9	82 ± 7	74 ± 7	91 ± 7	0.2 ± 0.0	13 ± 2	10 ± 1	99 ± 5	77 ± 3
***Npc1*** ^***-/-***^	**85**		**28.8 ± 0.7**	**45 ± 3** *	**311 ± 17**	**119 ± 10**	**1.4 ± 0.1**	**338 ± 12** **	**522 ± 15** *	**421 ± 17**	**291 ± 15** *
	**85**	**1 (21)**	26.1 ± 1.3	42 ± 3	1195 ± 142	75 ± 6	2.2 ± 0.5	229 ± 25	378 ± 12	430 ± 85	204 ± 30
	**85**	**5 (21)**	29.5 ± 1.4	53 ± 6	1455 ± 171^†^	80 ± 5	2.3 ± 0.1	225 ± 24	396 ± 31	453 ± 92	275 ± 21
	**85**	**1 (31)**	33.4 ± 0.2	53 ± 11	936 ± 86	82 ± 16	2.7 ± 0.0*	263 ± 25	448 ± 50	408 ± 15	239 ± 19
	**85**	**5 (31)**	31.0 ± 1.0	60 ± 4	1599 ± 54^††^	112 ± 3	2.2 ± 0.3	193 ± 4	310 ± 17	473 ± 54	256 ± 20
***Npc1*** ^***+/+***^	**110**		N.A.	107 ± 8	115 ± 24	85 ± 8	0.3 ± 0.0	17 ± 3	11 ± 3	N.A.	N.A.
***Npc1*** ^***-/-***^	**110**	**1 (21)**	24.2 ± 2.5	48 ± 2^‡^	1181 ± 170	73 ± 11	3.1 ± 0.3	264 ± 2	412 ± 6	382 ± 17	255 ± 32
	**110**	**5 (21)**	23.3 ± 3.8	58 ± 5	1381 ± 155	81 ± 2	3.7 ± 0.4	274 ± 28	477 ± 49	378 ± 78	193 ± 10
	**110**	**1 (31)**	25.5 ± 2.7	48 ± 4	1015 ± 139	65 ± 4	3.1 ± 0.2	275 ± 2	442 ± 10	341 ± 53	256 ± 35
	**110**	**5 (31)**	22.7 ± 1.7	65 ± 7	1572 ± 92^‡^	97 ± 19	3.8 ± 0.6	293 ± 10	474 ± 9	450 ± 16	300 ± 15

Levels of GalCer, GlcCer, Cer, GlcSph and gangliosides (GM3 and GM2), and activity of lysosomal enzymes β-hexosaminidase and β-glucuronidase in whole brain homogenates of *Npc1* ^*+/+*^, *Npc1* ^*+/-*^ and *Npc1* ^-/-^ treated and untreated 85- and 110-day-old (BALB/c) mice. Data (*n* = 6–12 per group) were analyzed using the Kruskal-Wallis test with *post hoc* Dunn’s multiple comparison test.

* *P* < 0.05 and

** *P* < 0.01 from untreated *Npc1* ^+/+^ (85-day-old)

^†^ *P* < 0.05 and

^††^ *P* < 0.01 from untreated *Npc1* ^+/-^ (85-day-old)

^‡^ *P* < 0.05 from untreated *Npc1* ^+/+^ (110-day-old)

Lipid analysis of dissected cerebella gave very similar results to total brain (S7 Fig). Ceramide (*P* < 0.05), GlcCer (*P* < 0.05) and GlcSph (*P* < 0.001) levels were increased in the cerebellum of 75-day-old untreated *Npc1* ^*-/-*^ mice compared to age-matched *wt* animals. In end-stage (115-day-old) *Npc1* ^*-/-*^ mice, treatment did not affect ceramide levels and aggravated elevations in GlcCer and GlcSph. GalCer was reduced in cerebellum of *Npc1* ^*-/-*^ mice.

As previously reported [12], lysosome markers (β-hexosaminidase and β-glucuronidase) were elevated in brain of *Npc1* ^*-/-*^ mice as a consequence of lysosomal hypertrophy. Iminosugar-treatment did not normalize these markers (Table 1). Noteworthy, the levels of cholesterol, GSLs, GlcSph, and lysosome markers were similar in 85-day-old brains of *Npc1* ^-/-^/*Gba2* ^-/-^ mice compared to those in brains of age-matched iminosugar-treated *Npc1* ^*-/-*^ animals (Table 2). Hence, pharmacological or genetic abrogation of GBA2 enzymatic activity while ameliorating neurological manifestation does not concomitantly correct the lysosomal hypertrophy or the accumulation of GSLs in the central nervous system of *Npc1* ^*-/-*^ mice.

**Table 2:** Genetic ablation of *Gba2* does not correct lysosomal defects.

	Cholesterol (μmol/g ww)	Glycosphingolipids (nmol/g ww)						Activity (% *wt*)
		GalCer (% *wt*)	GlcCer (% *wt*)	Cer (% *wt*)	GlcSph	GM3	GM2	β-hexo
***Npc1*** ^**-/-**^ **/*Gba2*** ^**-/-**^	13.8 ± 0.7	45 ± 3**	1096 ± 25***	89 ± 9	4.5 ± 0.5***	327 ± 16	335 ± 20*	333 ± 13
***Npc1*** ^**-/-**^ **/*Gba2*** ^**+/-**^	14.7 ± 0.7	53 ± 3	312 ± 10*	115 ± 7	2.6 ± 0.3*	402 ± 42**	384 ± 37**	352 ± 18
***Npc1*** ^**-/-**^ **/*Gba2*** ^**+/+**^	14.1 ± 0.5	46 ± 4**	257 ± 22	102 ± 8	2.0 ± 0.3	299 ± 27	310 ± 23	364 ± 9
***Npc1*** ^**+/+**^ **/*Gba2*** ^**+/+**^	16.2 ± 0.4	100 ± 6	100 ± 8	100 ± 5	0.3 ± 0.0	3 ± 2	1 ± 0	100 ± 5

Levels of GalCer, GlcCer, Cer, GlcSph and gangliosides (GM3 and GM2), and activity of lysosomal enzymes β-hexosaminidase in whole brain homogenates of mice resulting from the crossing of *Gba2* ^-/-^ mice with *Npc1* ^-/-^ mice. Data (*n* = 5–6 per group) were analyzed using the Kruskal-Wallis test with *post hoc* Dunn’s multiple comparison test.

* *P* < 0.05,

** *P* < 0.01 and

*** *P* < 0.001 from *Npc1* ^+/+^/*Gba2* ^+/+^

## Discussion

In the present study we examined the potential role of the non-lysosomal glucosylceramidase GBA2 in neurological manifestations in *Npc1* ^-/-^ mice. Firstly, we established that GBA2 activity is indeed abnormally high in the brain of *Npc1* ^-/-^ mice. This was indicated by analysis of brain using ABP–labelling of GBA2, as well as by detection of GBA2 in brain lysates by Western blot and the measurement of enzymatic activity. The enzyme was also found to be particularly abundant in PCs. Even though we did not observe a striking reduction in GBA activity in the *Npc1* ^*-/-*^ CNS, as assayed *in vitro* with ABP 1 and artificial substrate, the accumulation of GBA-substrates (GlcCer and GlcSph) points to an impairment of the enzyme *in vivo*. Secondly, we examined the outcome of introducing a genetic loss of GBA2 in *Npc1* ^-/-^ mice. This ablation led to a significant increase in lifespan and amelioration of motor coordination, reflected by prolonged survival of PCs. Next, we investigated whether the loss of the GBA2 protein as such or the absence of its activity exerted the beneficial effect. Employing extremely potent GBA2 inhibitors (IC_50_ values < 5 nM), we observed that administration of as little as 25 μg per mouse daily led to similar beneficial responses as observed with genetic loss of GBA2, such as increased maximal body weight, delayed PC loss and delayed onset of motor deficits. The genetic background of mice is known to influence severity of disease manifestation in *Npc1* ^-/-^ animals [32]. Pharmacological inhibition of GBA2 in BALB/c *Npc1* ^-/-^ mice led to a relatively larger increase in life span (27–38%, Fig 3B) than genetic loss of GBA2 in *Npc1* ^-/-^ mice in mixed background (15% lifespan increase, Fig 2B). Whether this difference can be accounted to the different genetic backgrounds or points to an additional target for the iminosugars next to GBA2 is presently unclear. It has to be stressed that low nanomolar concentrations of iminosugars were applied at which no other enzymes are known to be inhibited.

Our combined findings point out that GBA2 plays a role in the early neuropathology of NPC disease in mice. Our study also reveals that pharmacological inhibition of GBA2 offers an avenue to ameliorate neurological manifestation of NPC, in particular to delay the loss of motor coordination.

Our study led to the unexpected finding that while genetic or pharmacological reduction of GBA2 activity exerts beneficial effects in *Npc1* ^*-/-*^ mice this is not accompanied by reduction of the ganglioside accumulation and lysosomal hypertrophy in the brains of these animals. Clearly, reduction of GBA2 does not entirely prevent neuropathology, but rather delays this process. It seems likely that for complete prevention of disease, the primary underlying defect at the level of lysosomes should be resolved and further prevented. A combination therapy with a low dose of cyclodextrins might offer good prospects in this regard [35]. Nevertheless, a significant delay in loss of motor coordination in NPC patients through GBA2 activity, as observed in the mouse model, would be of great clinical value. In addition, our findings suggest that extra-lysosomal processes during lysosomal deficiency contribute to disease manifestation, a notion prior formulated by other researchers [8,36].

The molecular mechanism underlying the detrimental effect of cytosolic GBA2 activity on NPC pathology is presently not known. It might be envisioned that over-production by GBA2 of cytosolic ceramide and/or sphingosine from GlcCer and GlcSph is harmful, for example through induction of apoptosis or other cell death pathways in neuronal cells, particularly those with high GBA2 activity such as PCs. Of note, excessive formation of ceramide by GBA2 has recently been reported to promote apoptosis in human melanoma cells [37]. Detection of excessive cytosolic ceramide in NPC mice cerebella would require high-resolution *in situ* mass-spectrometry analysis, which at present is technically challenging. Our study renders no indication that the slightly elevated GlcSph in brains of *Npc1* ^*-/-*^ mice causes PC loss. The beneficial pharmacological inhibition or genetic ablation of GBA2 both led to a small further increase in GlcSph. At present it cannot be excluded that GBA2 has another unknown activity leading to formation of toxic compounds, for example through trans-glycosylation of other lipid species.

A currently approved drug for NPC disease is N-butyl-1-deoxynojirimycin (Zavesca, Actelion). N-alkylated iminosugars such as Zavesca inhibit GlcCer synthase (GCS), the enzyme catalyzing the first step in the biosynthesis of GSLs [38]. Zavesca has been found to ameliorate GD in mildly affected type 1 patients and was therefore approved for treatment of this condition [39]. Since excessive gangliosides have been postulated to contribute to neuropathology in NPC disease [40], the effect of Zavesca on NPC was studied in mouse models. At high concentrations Zavesca was found to reduce inflammation and ganglioside accumulation in the CNS of NPC mice, concomitantly delaying the onset of neuromotor symptoms [40]. In humans, treatment with this iminosugar was reported to stabilize and in some cases improve neurological aspects in NPC patients [41], leading to its registration as “orphan drug” for NPC disease in Europe. It is relevant to point out in this context, that iminosugars like Zavesca are not specific GCS inhibitors. They also inhibit the hydrolases involved in the degradation of GlcCer to ceramide: GBA and GBA2 [4,6]. Zavesca inhibits GBA2 with much higher affinity than GCS (IC_50_ = 230 nM versus IC_50_ = 50,000 nM, respectively) [42]. Inhibition of GBA2 activity may therefore partially underlie the observed beneficial effect of Zavesca in NPC patients. The inhibitors used in our study, AMP-DNM and L-ido-AMP-DNM, are far more specific GBA2 inhibitors than Zavesca. Oral administration of AMP-DNM was reported to increase survival of NPC mice and Sandhoff disease mice [34,43]. The beneficial effects of AMP-DNM in the present study occurred at drug concentrations at which effective inhibition of GCS is highly unlikely, whereas complete inhibition of GBA2 activity occurs. For further optimization of iminosugar-type drugs for NPC and comparable disease conditions it will be key to establish firmly whether inhibition of GBA2 or that of GCS, or even both enzymes, is required to exert an optimal clinical response.

In conclusion, GBA2 inhibition suffices to significantly increase lifespan of *Npc1* ^*-/-*^ mice despite persistent lysosomal abnormalities. Evidently, reduction of this enzymatic activity in *Npc1* ^*-/-*^ mice, or even genetic absence of the enzyme, does not completely prevent neurodegeneration. Nevertheless, our results suggest that pharmacological inhibition of GBA2 might result in a clinically significant delay of neurological complications. Given their advantageous inhibitory profile GBA2 inhibitors AMP-DNM (MZ-21) and *l*-ido-AMP-DNM (MZ-31) may serve as lead components for drug development for NPC.

## Supporting Information

S1 ARRIVE Checklist (PDF)

S1 Fig GBA and GBA2 activity in total brain and cerebellum of *Npc1* ^*+/+*^, *Npc1* ^*+/-*^ and *Npc1* ^-/-^ mice. Quantification of scanned band intensity of ABP 1-fluorescently labelled (slab gel) and immuno-probed (Western blot) GBA and GBA2 in brain **(A)** and cerebellum **(C)** homogenates of *Npc1* ^+/+^, *Npc1* ^+/-^ and *Npc1* ^-/-^ mice (*see* Fig 1B and 1D). Intensity (arbitrary units) was normalized to α-tubulin and expressed relative to *wt* (*Npc1* ^+/+^) control. GBA and GBA2 enzymatic activities (assayed with 4MU-β-D-Glc substrate) in brain **(B)** and cerebellum **(D)** homogenates of *Npc1* ^*+/+*^, *Npc1* ^*+/-*^ and *Npc1* ^-/-^ mice (*see* Fig 1C and 1E).
(TIF)

S2 Fig GBA2/GBA activity ratio in brain areas of *Npc1* ^*+/+*^, *Npc1* ^*+/-*^ and *Npc1* ^-/-^ mice. Ratio of GBA2 and GBA enzymatic activities (assayed with 4MU-β-D-Glc substrate) in homogenates of dissected pons-medulla, cortex, hippocampus, olfactory bulb, hypothalamus and remaining brain (rest) of 75-day-old *Npc1* ^*+/+*^, *Npc1* ^*+/-*^ and *Npc1* ^-/-^ mice.
(TIF)

S3 Fig Natural history of GBA and GBA2 protein expression in the cerebellum. Sagittal cerebellar sections of 40-, 60- and 80-day-old *Npc1* ^*+/+*^ and *Npc1* ^*-/-*^ mice were single immunostained with anti-calbindin D-28K and anti-GFAP antibodies. Staining patterns in the anterior cerebellum indicate progressive loss of calbindin D-28K positive Purkinje cells and concomitant increase of GFAP positive astrogliosis in *Npc1* ^*-/-*^ mice (top panels; scale bar = 500 μm). Sagittal cerebellar sections of 40-, 60- and 80-day-old *Npc1* ^*+/+*^ and *Npc1* ^*-/-*^ mice were double immunostained with anti-GBA and anti-GBA2 antibodies. For the central cerebellar zones, in the region where only few calbindin D-28K positive Purkinje cells remain in *Npc1* ^*-/-*^ mice at day 80, staining patterns of anti-GBA and anti-GBA2 are each displayed separately (small bottom panels; scale bar = 100 μm) and as composite image (large bottom panels; scale bar = 100 μm). The arrows indicate Purkinje cells.
(TIF)

S4 Fig Quantification of ABP 1-labelling and immunoprobing in brain homogenates of end-stage *Npc1* ^-/-^ and *Gba2* ^-/-^ crossed animals. Quantification of scanned band intensity of ABP1-fluorescently labelled (slab gel) GBA and GBA2 and immuno-probed (Western blot) GBA2 in brain homogenates of end-stage *Npc1* ^-/-^ and *Gba2* ^-/-^ crossed animals (*see* Fig 2C). Intensity (arbitrary units) was normalized to α-tubulin and expressed relative to *wt* (*Npc1* ^+/+^/*Gba2* ^+/+^) control.
(TIF)

S5 Fig Quantification of ABP 1-labelling and immunoprobing in brain homogenates of *Npc1* ^*+/+*^, *Npc1* ^*+/-*^ *and Npc1* ^-/-^ iminosugar-treated mice. Quantification of scanned band intensity of ABP1-fluorescently labelled (slab gel) GBA and GBA2 and immuno-probed (Western blot) GBA2 in brain homogenates of *Npc1* ^*+/+*^, *Npc1* ^*+/-*^ *and Npc1* ^-/-^ iminosugar-treated and untreated 85- and 110-day-old mice (*see* Fig 3C). Intensity (arbitrary units) was normalized to α-tubulin and expressed relative to *wt* (*Npc1* ^+/+^) control.
(TIF)

S6 Fig Microglia and activated macrophage markers in *Npc1* ^-/-^ untreated and iminosugar-treated mice. (A) Sagittal cerebellar sections of 85-day-old *Npc1* ^+/+^ and *Npc1* ^-/-^ untreated mice and treated with 1 mpk MZ21 stained with HE and immunostained with anti-Iba-1 and -F4/80 antibodies. Scale bar = 100 μm. (B) Klüver-PAS staining of sagittal cerebellar sections of 85-days old *Npc1* ^+/+^ and *Npc1* ^-/-^ untreated mice and treated with 1 mpk MZ21. Scale bar = 50 μm.
(TIF)

S7 Fig GSL levels in cerebella of iminosugar treated mice. Levels of ceramide, GlcCer, GalCer and GlcSph in dissected cerebella of *Npc1* ^*+/+*^, *Npc1* ^*+/-*^ *and Npc1* ^-/-^ mice untreated (75 days of age) and treated with 5 mpk MZ-21 (115 days of age).
(TIF)

S8 Fig Validation of immunostainings. (A) Single indirect immunostaining using rabbit-anti-GBA2 primary antibody, HRP-conjugated Goat-anti-Rabbit secondary antibody, and DAB as substrate did detect Purkinje cells (PCs) in cerebellum of *Npc1* ^+/+^/*Gba2* ^+/+^ mice and did not label PCs nor any other structures in cerebellum of *Npc1* ^-/-^/*Gba2* ^-/-^ mice. Haematoxylin was used as counterstain. (B) Cerebellum of *Npc1* ^+/-^/*Gba2* ^-/-^ mice, which do not suffer from NPC disease and maintain presence of PCs as shown in haematoxylin and eosin (HE) stained sections, is not stained by anti-GBA2. (C) Double immunostaining directed against either first GBA and then GBA2 or first GBA2 and then GBA on *Npc1* ^+/+^/*Gba2* ^+/+^ cerebellum showed detection of GBA2 in PCs, both in the cell body and in the dendrites, and of GBA mainly in a punctuated pattern in the PC cell body. Background staining with secondary AP- and HRP-conjugated antibodies or with substrates VectorBlue and AMEC-Red was negligible. Methyl Green was applied as counterstain. (D) Double immunostaining with anti-GBA and anti-GBA2 revealed only binding of anti-GBA on PCs of *Npc1* ^-/-^/*Gba2* ^-/-^ mice.
(TIF)

S1 Table Age of euthanasia of individual *Npc1* ^-/-^ and *Gba2* ^-/-^ crossed animals. (XLS)

S2 Table Detailed statistical analysis of Kaplan-Meier survival plot of *Npc1* ^-/-^ and *Gba2* ^-/-^ crossed animals. (XLS)

S1 Text (DOC)
