# Supplementary figures and images for: Reducing GBA2 Activity Ameliorates Neuropathology in Niemann-Pick Type C Mice
Source: PLoS One. 2015 Aug 14;10(8):e0135889. doi: 10.1371/journal.pone.0135889 (PMC4537125; doi:10.1371/journal.pone.0135889)

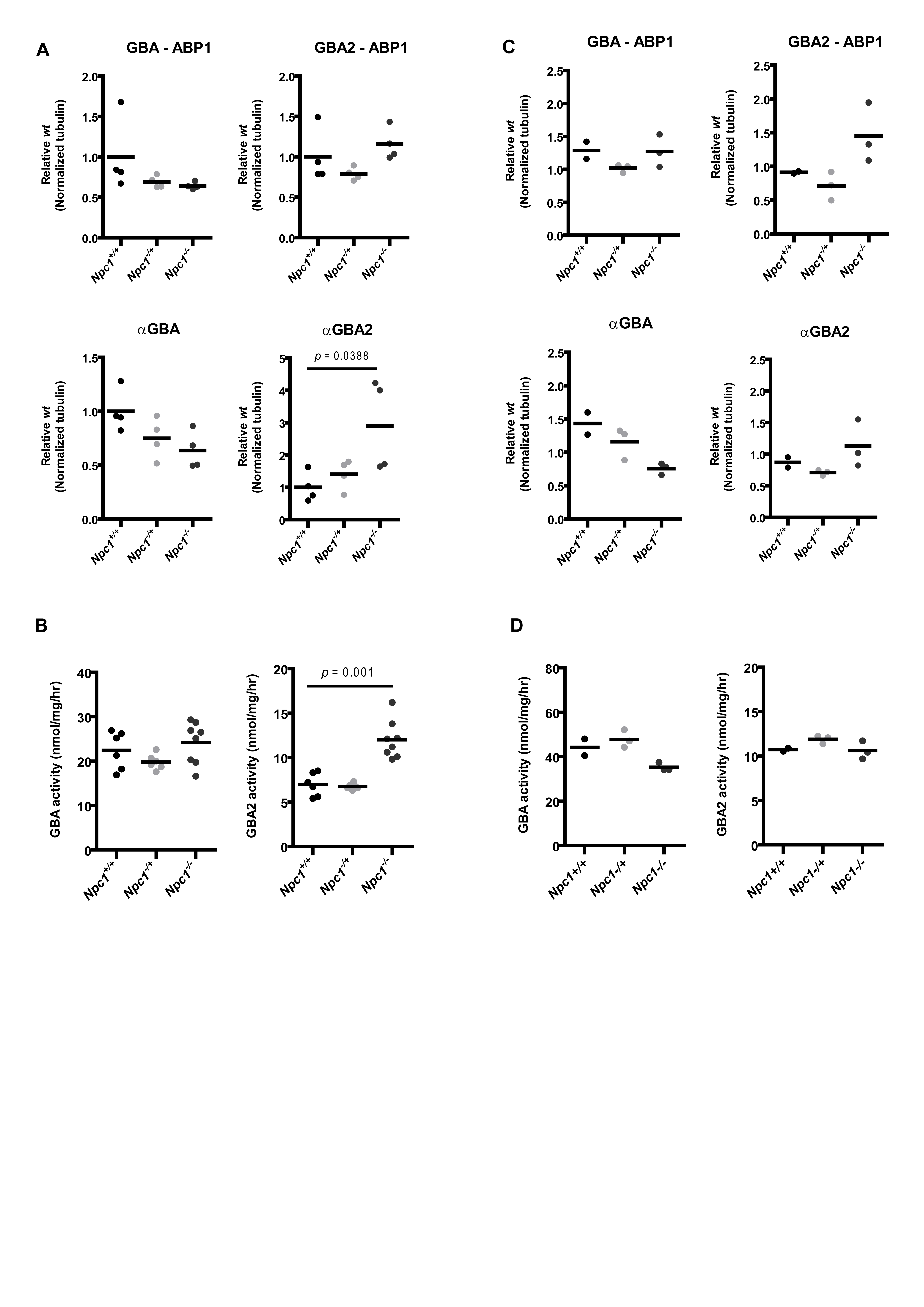

Supplement: S1 Fig — Quantification of scanned band intensity of ABP 1-fluorescently labelled (slab gel) and immuno-probed (Western blot) GBA and GBA2 in brain (A) and cerebellum (C) homogenates of Npc1 +/+, Npc1 +/- and Npc1 -/- mice (see Fig 1B and 1D). Intensity (arbitrary units) was normalized to α-tubulin and expressed relative to wt (Npc1 +/+) control. GBA and GBA2 enzymatic activities (assayed with 4MU-β-D-Glc substrate) in brain (B) and cerebellum (D) homogenates of Npc1 +/+, Npc1 +/- and Npc1 -/- mice (see Fig 1C and 1E). (TIF) [file pone.0135889.s002.tif]

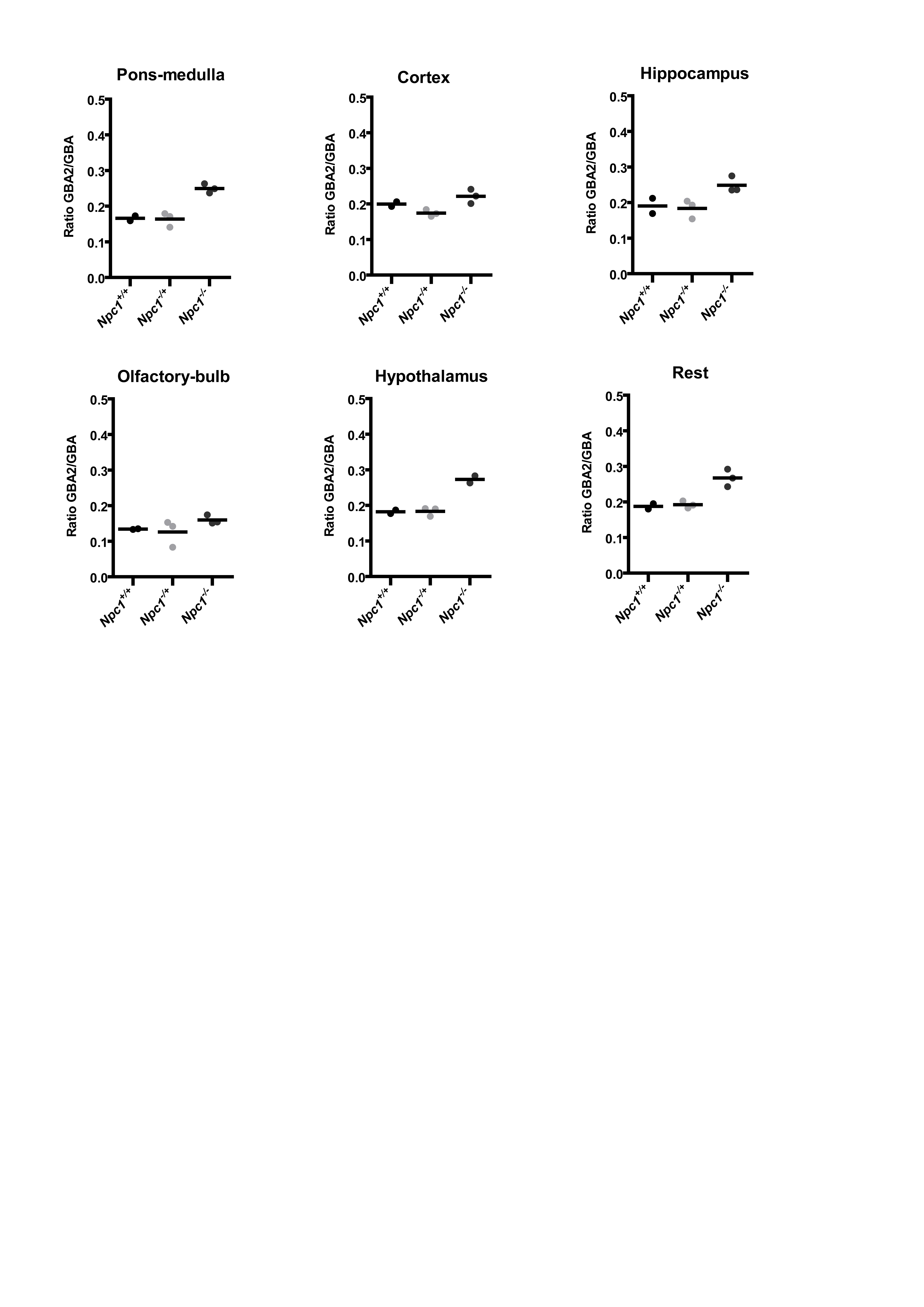

Supplement: S2 Fig — Ratio of GBA2 and GBA enzymatic activities (assayed with 4MU-β-D-Glc substrate) in homogenates of dissected pons-medulla, cortex, hippocampus, olfactory bulb, hypothalamus and remaining brain (rest) of 75-day-old Npc1 +/+, Npc1 +/- and Npc1 -/- mice. (TIF) [file pone.0135889.s003.tif]

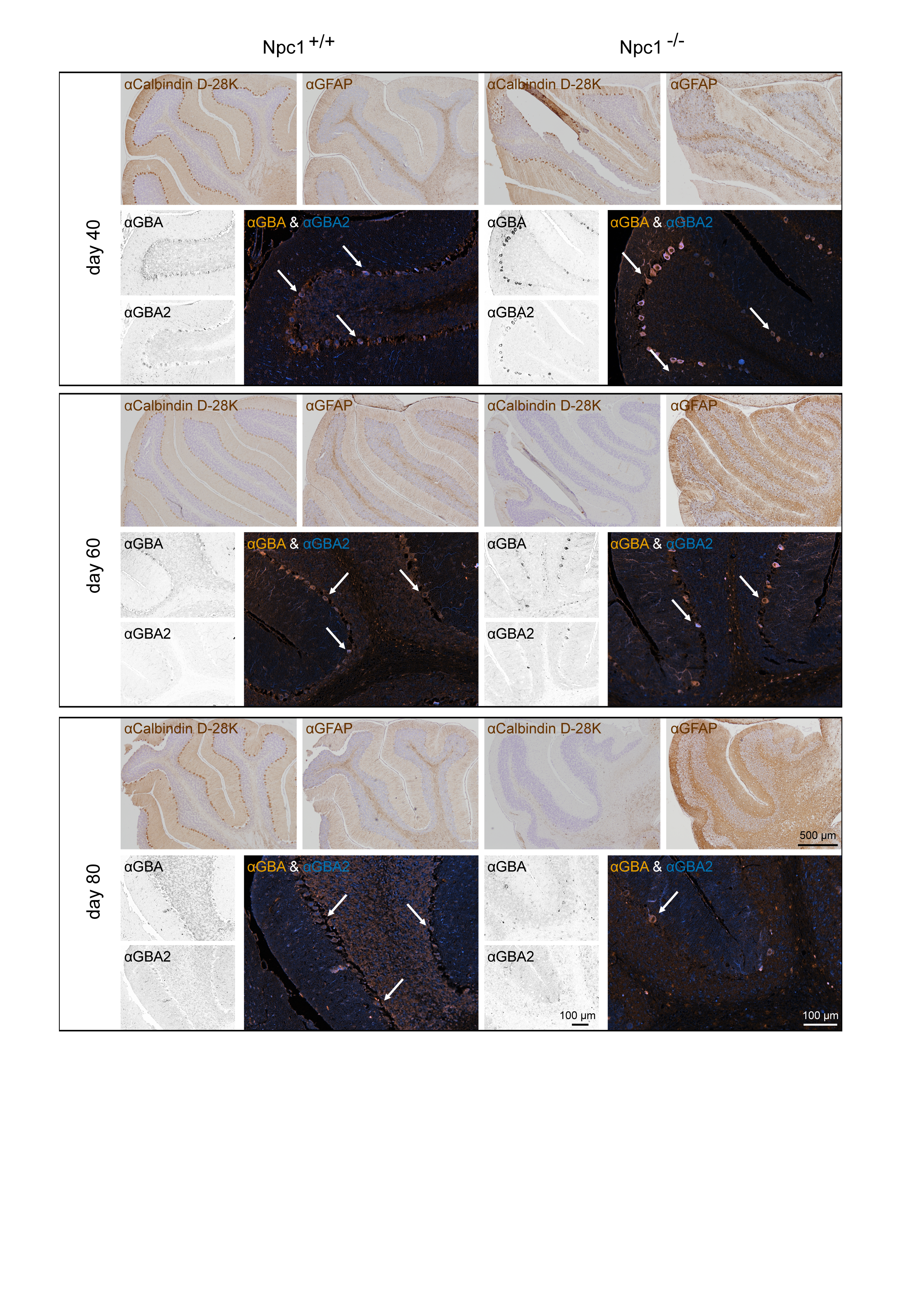

Supplement: S3 Fig — Sagittal cerebellar sections of 40-, 60- and 80-day-old Npc1 +/+ and Npc1 -/- mice were single immunostained with anti-calbindin D-28K and anti-GFAP antibodies. Staining patterns in the anterior cerebellum indicate progressive loss of calbindin D-28K positive Purkinje cells and concomitant increase of GFAP positive astrogliosis in Npc1 -/- mice (top panels; scale bar = 500 μm). Sagittal cerebellar sections of 40-, 60- and 80-day-old Npc1 +/+ and Npc1 -/- mice were double immunostained with anti-GBA and anti-GBA2 antibodies. For the central cerebellar zones, in the region where only few calbindin D-28K positive Purkinje cells remain in Npc1 -/- mice at day 80, staining patterns of anti-GBA and anti-GBA2 are each displayed separately (small bottom panels; scale bar = 100 μm) and as composite image (large bottom panels; scale bar = 100 μm). The arrows indicate Purkinje cells. (TIF) [file pone.0135889.s004.tif]

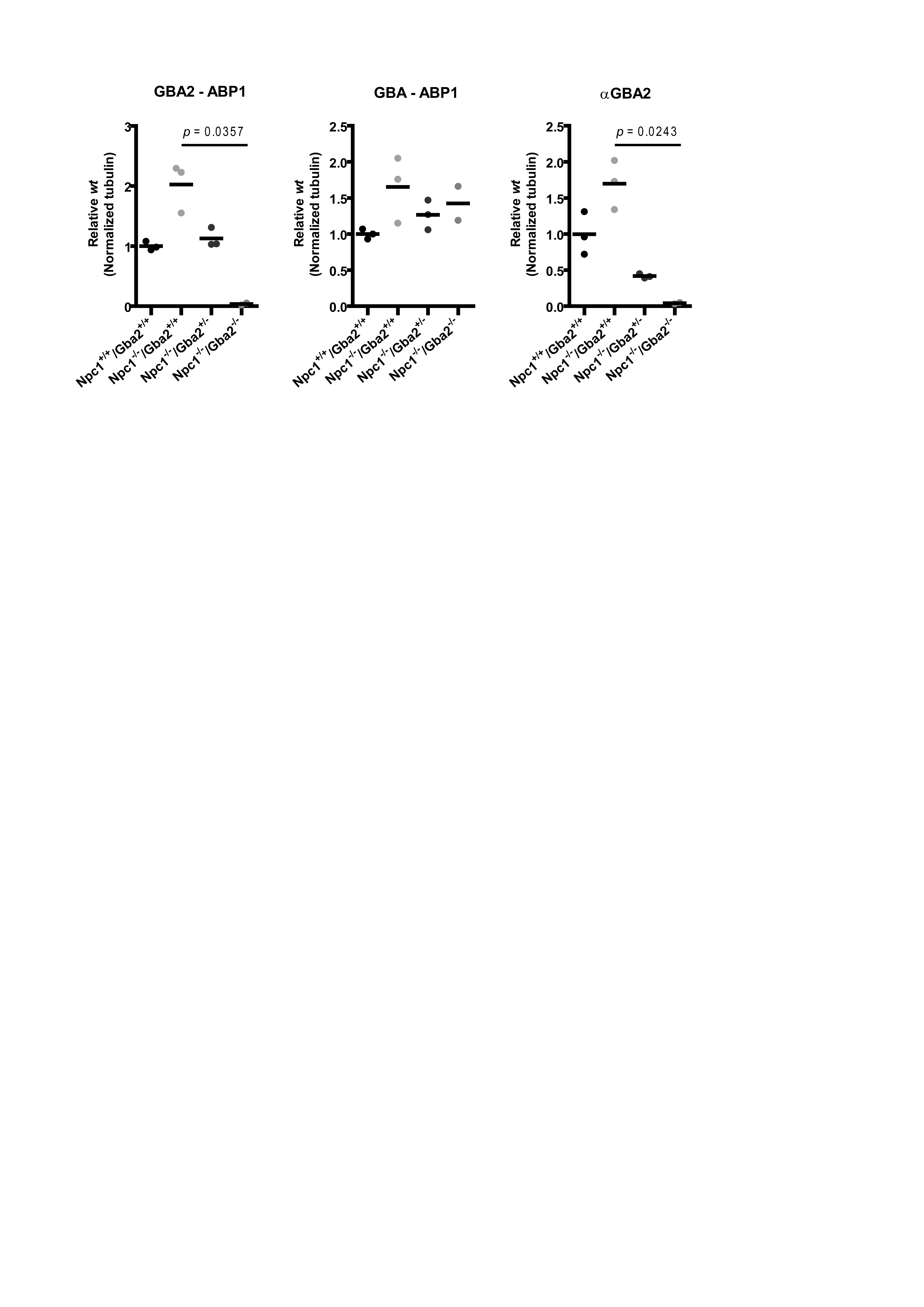

Supplement: S4 Fig — Quantification of scanned band intensity of ABP1-fluorescently labelled (slab gel) GBA and GBA2 and immuno-probed (Western blot) GBA2 in brain homogenates of end-stage Npc1 -/- and Gba2 -/- crossed animals (see Fig 2C). Intensity (arbitrary units) was normalized to α-tubulin and expressed relative to wt (Npc1 +/+/Gba2 +/+) control. (TIF) [file pone.0135889.s005.tif]

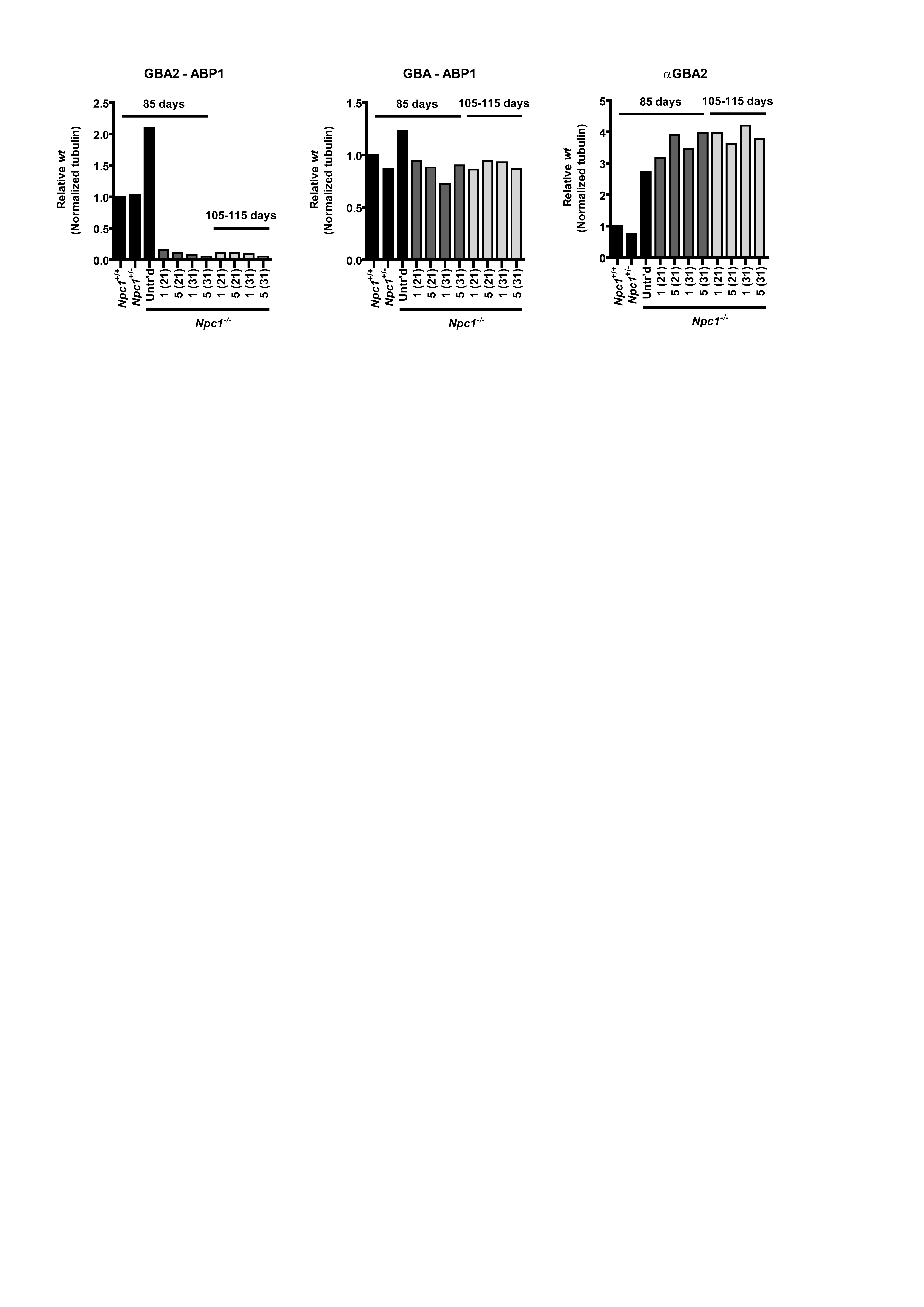

Supplement: S5 Fig — Quantification of scanned band intensity of ABP1-fluorescently labelled (slab gel) GBA and GBA2 and immuno-probed (Western blot) GBA2 in brain homogenates of Npc1 +/+, Npc1 +/- and Npc1 -/- iminosugar-treated and untreated 85- and 110-day-old mice (see Fig 3C). Intensity (arbitrary units) was normalized to α-tubulin and expressed relative to wt (Npc1 +/+) control. (TIF) [file pone.0135889.s006.tif]

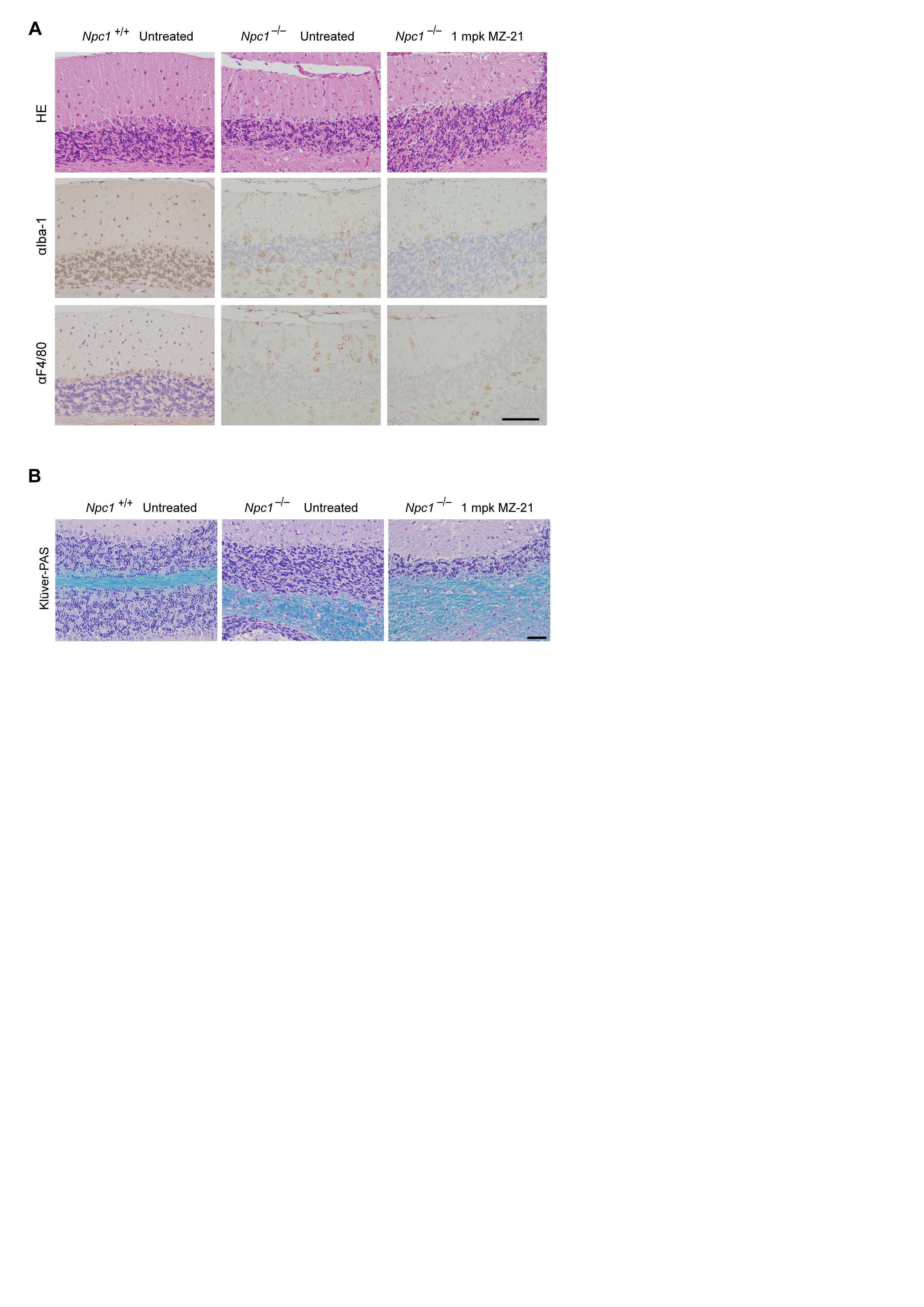

Supplement: S6 Fig — (A) Sagittal cerebellar sections of 85-day-old Npc1 +/+ and Npc1 -/- untreated mice and treated with 1 mpk MZ21 stained with HE and immunostained with anti-Iba-1 and -F4/80 antibodies. Scale bar = 100 μm. (B) Klüver-PAS staining of sagittal cerebellar sections of 85-days old Npc1 +/+ and Npc1 -/- untreated mice and treated with 1 mpk MZ21. Scale bar = 50 μm. (TIF) [file pone.0135889.s007.tif]

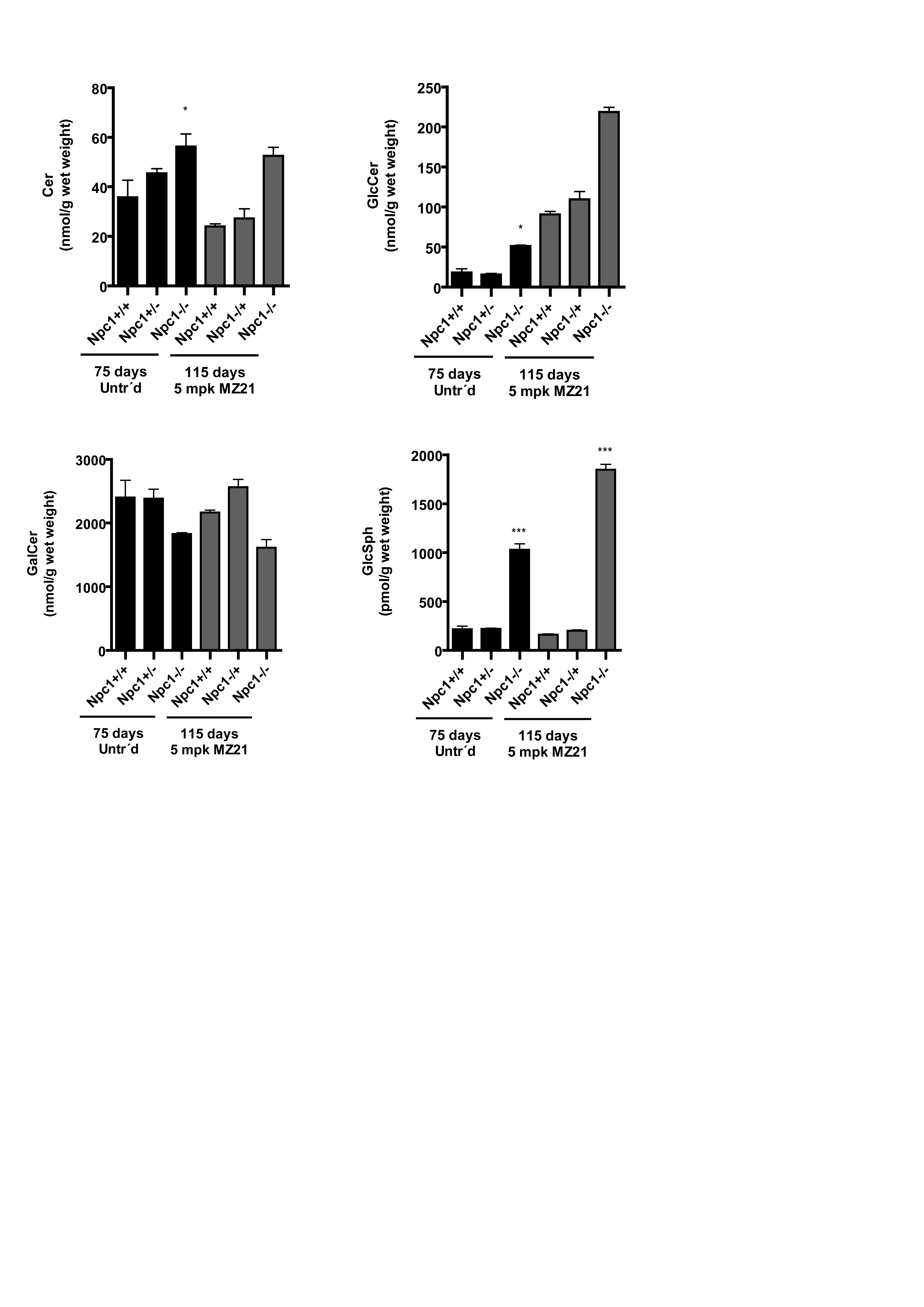

Supplement: S7 Fig — Levels of ceramide, GlcCer, GalCer and GlcSph in dissected cerebella of Npc1 +/+, Npc1 +/- and Npc1 -/- mice untreated (75 days of age) and treated with 5 mpk MZ-21 (115 days of age). (TIF) [file pone.0135889.s008.tif]

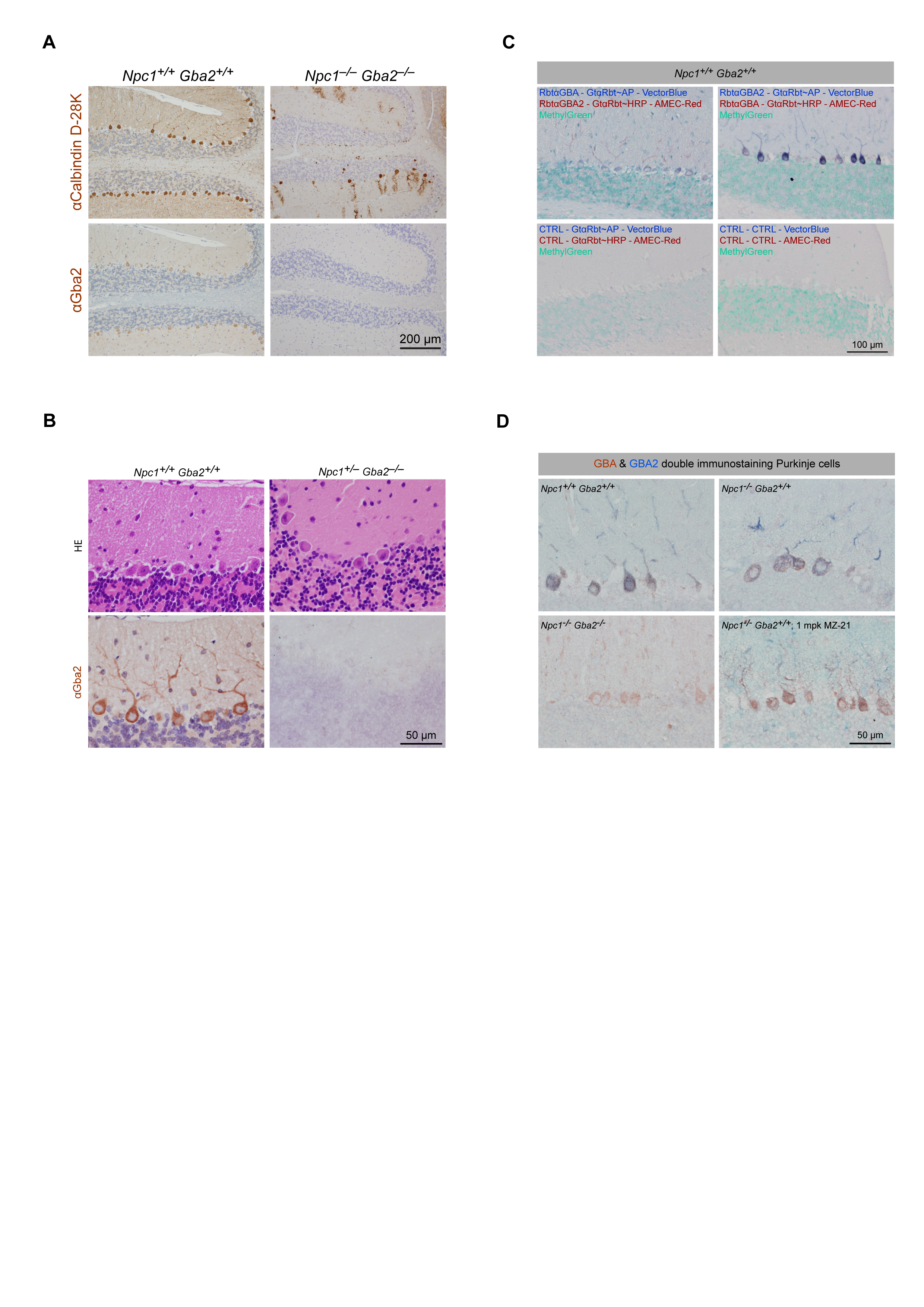

Supplement: S8 Fig — (A) Single indirect immunostaining using rabbit-anti-GBA2 primary antibody, HRP-conjugated Goat-anti-Rabbit secondary antibody, and DAB as substrate did detect Purkinje cells (PCs) in cerebellum of Npc1 +/+/Gba2 +/+ mice and did not label PCs nor any other structures in cerebellum of Npc1 -/-/Gba2 -/- mice. Haematoxylin was used as counterstain. (B) Cerebellum of Npc1 +/-/Gba2 -/- mice, which do not suffer from NPC disease and maintain presence of PCs as shown in haematoxylin and eosin (HE) stained sections, is not stained by anti-GBA2. (C) Double immunostaining directed against either first GBA and then GBA2 or first GBA2 and then GBA on Npc1 +/+/Gba2 +/+ cerebellum showed detection of GBA2 in PCs, both in the cell body and in the dendrites, and of GBA mainly in a punctuated pattern in the PC cell body. Background staining with secondary AP- and HRP-conjugated antibodies or with substrates VectorBlue and AMEC-Red was negligible. Methyl Green was applied as counterstain. (D) Double immunostaining with anti-GBA and anti-GBA2 revealed only binding of anti-GBA on PCs of Npc1 -/-/Gba2 -/- mice. (TIF) [file pone.0135889.s009.tif]
