# Supplementary material for: Reducing GBA2 Activity Ameliorates Neuropathology in Niemann-Pick Type C Mice
Source: PLoS One. 2015 Aug 14;10(8):e0135889. doi: 10.1371/journal.pone.0135889 (PMC4537125; doi:10.1371/journal.pone.0135889)
Supplement: S1 Text — (DOC) [file pone.0135889.s012.doc]

**Supplemental Information**

**Supplemental Material and Methods**

***Quantification of band intensity.***

Quantification of gel scans was performed using National Institutes of Health (NIH) ImageJ software.

***Erasmus Ladder.***

We tested motor coordination of non-treated and iminosugar-treated *Npc1* mice using the Erasmus Ladder. Details on the device and its software have been published before [1–3]. In short, the Erasmus Ladder consists of a horizontal ladder between two shelter boxes, each equipped with an LED spotlight in the roof and two pressurized air outlets in the back (http://www.noldus.com/animal-behavior-research/products/erasmusladder). The light and air stimuli serve as cues controlling the moment of departure of the mice. The ladder itself has 37 rungs on each side and each rung is equipped with a custom-made pressure sensor that is continuously monitored, while mice are walking on the rungs. Moreover, each rung can be displaced vertically following a command from the control system so as to study the reaction of the mouse to appearance of an unexpected obstacle following a preceding tone. In the standard position even-numbered rungs are elevated by 6 mm on one side, whereas odd-numbered rungs are elevated on the other side, together creating a left/right alternating pattern. Both the recording and stimulating parts of the setup are controlled by software written in LabView (National Instruments, Austin, TX, USA) operating at a fixed cycle of 2 ms. All data collected are stored in a relational database (MySQL, Oracle, Redwood Shores, CA, USA) and subsequently processed and analyzed off-line using custom-written software in LabView and Python (Python Software Foundation, Beaverton, OR, USA) as well as SPSS (IBM Sorporation, Armonk, NY, USA).

For the current study we followed a modified version of a previously developed protocol [3]. In principle, if health status permitted, each mouse had to perform 1 daily session during 5 days at the age of 6 weeks and 1 daily session during 2 days at the age of 9 and 10 weeks. The sessions for the 6 weeks-old stage included in consecutive order 3 non-perturbed sessions so as to adjust to the apparatus, 1 fix-obstacle session during which rung number 19 remained always elevated by 18 mm (i.e. 12 mm above the walking path), and 1 paired session in which the unconditioned stimulus (i.e. elevated rung) was provided at a random location on the right side 200 ms following the onset of the conditioned stimulus (i.e. a tone), which in turn depended on the predicted trajectory of the mouse (for details see [1]). The sessions for the 9 and 10 weeks-old stages included 1 non-perturbed session followed by 1 fix-obstacle session. During all sessions mice had to walk back and forth between the 2 shelter boxes, and during all sessions, which always included 45 trials, we took step time as the main outcome parameter, which was defined as the time (in ms) that elapses between the onsets of two consecutive touches on the rungs.

***Immnohistochemistry.***

Protocol as described in the “Materials and Methods” section with the following additions. Tissue sections were incubated with primary antibody being either polyclonal rabbit IgG anti-Iba-1 (#019-19741, 1:200; Wako Pure Chemical Industries, Osaka, Japan) or rat monoclonal IgG2b anti-F4/80 (MCA497GA, 1:500; AbD Serotec, Oxford, UK). Tissue sections were stained with Luxol fast blue according to Klüver in combination with periodic acid-Schiff (Klüver-PAS).

**Supplemental Results**

***Erasmus Ladder.***

To evaluate the effect of pharmacological inhibition of GBA2 on motor coordination we tested non-treated mice (*Npc1*^-/-^ n = 4; *Npc1^+/-^*n = 5; *Npc1^+/+^* n = 4) and iminosugar-treated mice (*Npc1*^-/-^ n = 6; *Npc1^+/-^*n = 6; *Npc1^+/+^* n = 6) on the Erasmus Ladder. At the end of the 6 weeks-old stage (i.e. the paired session) there was a significant effect of genotype in the step times of the non-treated group (non-treated *p* = 0.028), but not in those of the iminosugar-treated group (*p* = 0.750). Moreover, post-hoc analysis (LSD) indicated that non-treated *Npc1^-/-^* mice needed significantly longer time to make a single step than non-treated *Npc1^+/+^* or non-treated *Npc1^+/-^* (non-treated *Npc1^-/-^* vs. *Npc1^+/+^ p =* 0.022; non-treated *Npc1^-/-^* vs. *Npc1^+/-^ p =* 0.015) (see Fig. 3G). Similarly, at 9 weeks of age there was also a highly significant main effect of genotype in the step times of the group of non-treated animals (non-perturbed non-treated *p* = 0.001 and fix-obstacle non-treated *p* = 0.002), but less so in those of the iminosugar-treated animals (non-perturbed iminosugar-treated *p =* 0.056 and fix-obstacle iminosugar-treated *p* = 0.047). Further post-hoc analysis demonstrated that non-treated *Npc1^-/-^* showed significantly longer step times than control mice during the non-perturbed session (*Npc1^-/-^* vs. *Npc1^+/+^ p =* 0.001; *Npc1^-/-^* vs. *Npc1^+/-^ p =* 0.001) and the fix-obstacle session (*Npc1^-/-^* vs. *Npc1^+/+^ p =* 0.002; *Npc1^-/-^* vs. *Npc1^+/-^ p =* 0.001), whereas these differences were not observed for the iminosugar-treated animals during the fix-obstacle session (*Npc1^-/-^* vs. *Npc1^+/+^ p =* 0.060; *Npc1^-/-^* vs. *Npc1^+/-^ p =* 0.022) (Fig. 3G). A direct comparison between the step times of treated and non-treated *Npc1^-/-^* animals during the sessions of the 6 and 9 weeks stages also showed a significant difference (6 weeks *p* = 0.218; 9 weeks *p* = 0.016). After 9 weeks, non-treated *Npc1^-/-^* animals reached humane endpoint in that they showed substantially weight loss, generalized decreased grooming and severe ataxia, and according to the ethical protocol they had to be sacrificed. However, the health conditions of the iminosugar-treated *Npc1^-/-^* mice were sufficient to test them once more. At 10 weeks of age there was a significant main effect of genotype in the step times of the iminosugar-treated *Npc1^-/-^* mice (non-perturbed *p* < 0.001; fix obstacle *p* < 0.001). Indeed, post-hoc analysis (LSD) demonstrated that at 10 weeks of age iminosugar-treated *Npc1^-/-^* mice needed significantly longer time than the control mice to make a single step during the non-perturbed session (*Npc1^-/-^* vs. *Npc1^+/+^ p* < 0.001; *Npc1^-/-^* vs. *Npc1^+/-^ p* < 0.001) and the fix-obstacle session (*Npc1^-/-^* vs. *Npc1^+/+^ p* < 0.001; *Npc1^-/-^* vs. *Npc1^+/-^ p =* < 0.001) (Fig. 3G). Thus, as time progressed iminosugar-treated *Npc1^-/-^* mice also developed impaired motor coordination, but the onset of motor impairments was delayed by about four weeks.

**References**

1. Van Der Giessen RS, Koekkoek SK, van Dorp S, De Gruijl JR, Cupido A, Khosrovani S, et al. Role of olivary electrical coupling in cerebellar motor learning. Neuron. 2008;58: 599–612. doi:10.1016/j.neuron.2008.03.016

2. Vinueza Veloz MF, Buijsen RAM, Willemsen R, Cupido A, Bosman LWJ, Koekkoek SKE, et al. The effect of an mGluR5 inhibitor on procedural memory and avoidance discrimination impairments in Fmr1 KO mice. Genes Brain Behav. 2012;11: 325–31. doi:10.1111/j.1601-183X.2011.00763.x

3. Vinueza Veloz MF, Zhou K, Bosman LWJ, Potters J-W, Negrello M, Seepers RM, et al. Cerebellar control of gait and interlimb coordination. Brain Struct Funct. 2014;[Epub]. doi:10.1007/s00429-014-0870-1
